# Rigour of development of European Society of Cardiology, American College of Cardiology and American Heart Association guidelines over a 12-year period (2013–2024): a systematic review of guidelines

**DOI:** 10.1093/ehjqcco/qcae113

**Published:** 2025-01-20

**Authors:** Daniel A Gomes, Sanjali A C Ahuja, Yi Ting Yu, Robert English, Mahmood Ahmad, Mohammed Khanji, Pedro Adragão, Rui Providência

**Affiliations:** Department of Cardiology, Hospital de Santa Cruz, Unidade Local de Saúde de Lisboa Ocidental, Carnaxide 2790-134, Portugal; Barts and The London School of Medicine and Dentistry, Queen Mary University London, Turner St, London E1 2AD, UK; Barts and The London School of Medicine and Dentistry, Queen Mary University London, Turner St, London E1 2AD, UK; Barts and The London School of Medicine and Dentistry, Queen Mary University London, Turner St, London E1 2AD, UK; Department of Cardiology, Royal Free Hospital, Royal Free London NHS Foundation Trust, 10 Pond St, London NW3 2PS, UK; Barts Heart Centre, St Bartholomew's Hospital, Barts Health NHS Trust, London EC1A 7BE, UK; Newham University Hospital, Barts Health NHS Trust, Glen Road, Plaistow, London E13 8SL, UK; NIHR Barts Biomedical Research Centre, William Harvey Research Institute, Queen Mary University, London EC1A 7BE, UK; Department of Cardiology, Hospital de Santa Cruz, Unidade Local de Saúde de Lisboa Ocidental, Carnaxide 2790-134, Portugal; Department of Cardiology, Hospital da Luz, Lisbon 1500-650, Portugal; Barts and The London School of Medicine and Dentistry, Queen Mary University London, Turner St, London E1 2AD, UK; Newham University Hospital, Barts Health NHS Trust, Glen Road, Plaistow, London E13 8SL, UK; Institute of Health Informatics Research, University College London, 222 Euston Road, London NW1 2DA, UK

**Keywords:** AGREE II, Rigour, Methods, Guidelines, Systematic review, ESC, ACC/AHA

## Abstract

**Introduction:**

The European Society of Cardiology (ESC) and the American College of Cardiology/American Heart Association (ACC/AHA) regularly publish guidelines for the management of cardiovascular disease. By definition, a guideline should follow strict methodological criteria, and have a transparent, traceable, and reproducible development process. We aimed to assess the overall strength of the recommendations and rigour of methodological development in ESC and ACC/AHA guidelines.

**Methods and results:**

A systematic review of ESC and ACC/AHA guidelines published from 2013 to 2024 was conducted. Documents class of recommendation (COR) and level of evidence (LOE) of recommendations were included. For each document, data regarding citation count (ISI and Scholar), and COR and LOE of the recommendations were extracted. Guidelines were assessed for rigour of methodological development using the Appraisal of Guidelines for Research & Evaluation II (AGREE II) instrument. Among the 76 included guidelines, the average citation-per-year was 344 (ISI) and 681 (Scholar). Forty-nine per cent of the recommendations were classified as COR I (strong recommendations), while 46% were based solely on expert opinion (LOE C). The overall AGREE II methodology domain score was 29 ± 6 (range 7–56), with the lowest performance for the domains of systematic search of evidence, use of pre-defined criteria for selecting the evidence and external review. Both the strength of the recommendations and rigour of development showed a stable trend over the past 12 years. ACC/AHA guidelines followed more rigorous development methods compared with ESC (AGREE II 36 ± 3 vs. 24 ± 3).

**Conclusions:**

Clinical guidelines from the main European and American cardiovascular societies are highly cited but show significant limitations in methodological rigour.

## Graphical Abstract

**Graphical Abstract:**
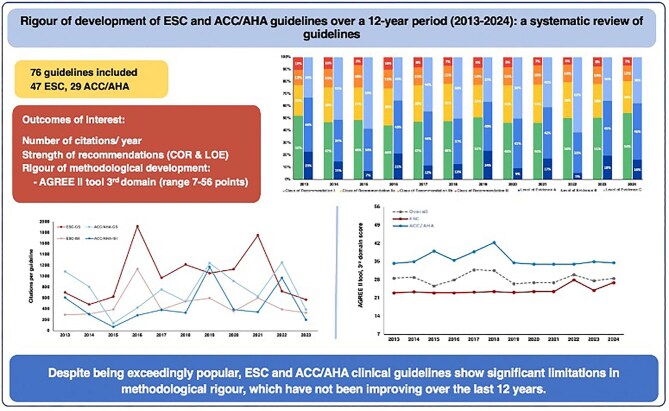

Key Learning Points **What is already known:**

- The European Society of Cardiology (ESC) and the American College of Cardiology/American Heart Association (ACC/AHA) regularly publish guidelines that are considered among the most important for the management of cardiovascular disease.
- However, no previous study has systematically assessed the quality of the main cardiovascular societies’ guidelines.

**What this study adds:**

- Our findings suggest that, although exceedingly popular, there is considerable room for improvement in the rigour of the development of most ACC/AHA and ESC guidelines.
- It is hoped that these results will encourage guideline authors and societies to deliver higher-quality recommendations, ultimately providing clinicians with the best information to make informed decisions.

## Introduction

A well-defined, transparent, and reproducible search strategy, along with clear criteria for evidence selection, is essential for developing evidence-based clinical guidelines.^1^ From a methodological perspective, a clinical practice guideline must provide clear proof that a systematic search strategy and other systematic review steps are performed.^2^ If these criteria are not met, the document should be referred to as a ‘guidance document’ rather than a ‘clinical guideline’.^2^

The AGREE Collaboration (Appraisal of Guidelines, Research and Evaluation) is an international consortium of researchers and guideline developers that created a tool to assess the quality of guideline development.^1,3^ The AGREE II instrument comprises 23 items categorized into 6 domains: I—scope and purpose, II—stakeholder involvement, III—rigour of development, IV—clarity of presentation, V—applicability, and VI—editorial independence.^1,3^ In areas such as oncology, only a small portion of guidelines have been informed by scientific evidence identified through adequate systematic search methods.^4^ Furthermore, more than 20 years ago, an overview showed that nearly 90% of guidelines did not report any information on the systematic search, with a small increase observed over the 90s (from 2% to 18%).^5^ Quality of practice guidelines is therefore a matter of concern, as lack of rigorous criteria can undermine guideline credibility and lead to harm to patients if the wrong recommendations are put into practice.^5^

European Society of Cardiology (ESC) and American College of Cardiology/American Heart Association ACC/AHA guidelines are considered to be amongst the most important cardiovascular disease guidelines in medicine and are used by physicians worldwide for assistance in clinical decision.^6–8^ For each recommendation there is a class of recommendation and a level of evidence (LOE). In both ESC and ACC/AHA guidelines, four classes of recommendation and three LOE are provided: Class I, Class IIa, Class IIb, and Class III; and LOE A (recommendation based on multiple randomized clinical trials (RCTs) or a single large RCT), LOE B (observational studies or a single RCT), and LOE C (expert opinion) (see Supplementary material online, *Table S1*).

The aims of this study were to determine (1) the rigour of the development of clinical guidelines from the main scientific cardiovascular societies in Europe and America over a 12-year period, as assessed by the third domain of the AGREE II tool; (2) the LOE underlying the different recommendations and the overall recommendations’ strength; and (3) guideline influence as measured through annual citation count.

## Methods

This systematic review was conducted in accordance with the Preferred Reported Items for Systematic Reviews and Meta-analyses (PRISMA) guidelines.^9^ The protocol was registered in the international prospective register of systematic reviews (PROSPERO CRD42024420147).

### Search strategy and selection criteria

We performed a systematic search of guidelines developed by the main European and American cardiovascular societies (ESC, ACC, and AHA) published from January 2013 to September 2024. The following official libraries were used: Clinical Practice Guidelines, ESC (available at https://www.escardio.org/Guidelines/Clinical-Practice-Guidelines); Library of Guidelines and Clinical Documents, ACC (available at https://www.acc.org/guidelines); and Professional Heart Daily, Guidelines and Statements, AHA (available at https://professional.heart.org/en/guidelines-and-statements). We also performed manual searches in PubMED using the terms ‘guidelines’ AND (‘ACC’ OR ‘AHA’ OR ‘ESC’) to assess for potential titles not in those libraries. Any retrieved documents including information and recommendations regarding diagnosis, work-up, management, treatment, or follow-up of cardiovascular conditions were considered potentially eligible. Only comprehensive guidelines that included recommendations organized by class of recommendation and LOE separated and highlighted from the rest of the text were included for this systematic review. To ensure appropriate representativeness of the evidence base for a given domain, only scientific documents reporting ≥20 summarized recommendations were considered. Expert consensus documents, performance measures, and appropriateness criteria were not included.

All records were manually screened, and duplicate entries (same guideline published by different cardiovascular societies) were removed. Each title was searched in PubMED to determine how many journals each guideline was published in. When the same guideline developer published different formats (summary, short or full versions), or in different journals, we included the most recent full version published in the scientific society highest impact factor journal. Additionally, manual searches in Google Scholar and ISI Web of Science were conducted through 1 August 2024 for determining the number of versions and citations for each document (total and adjusted by number of years since publication). For documents with multiple versions (summary and full/extended version) and publication in multiple journals, all available results were summed.

Searches were conducted by four authors working in pairs (D.A.G., S.A.C.A., Y.T.Y., and R.E.) and were validated by the senior author (R.P.). Full agreement for inclusion was required and disagreements were resolved via discussion or through the involvement of a third referee (R.P.).

### Data extraction

The following data were abstracted from all documents and double-checked by an independent reviewer (R.P.): (i) guideline characteristics: year of publication, title/cardiovascular condition of interest, and developing scientific cardiovascular society; (ii) guideline methodology: systematic methods for evidence search, selecting evidence, assessment on strengths and limitations of the body of evidence, methods for formulating the recommendations, consideration of health benefits, side effects and risks when formulating the recommendations, existence of a link between the recommendations and the supporting evidence, information on external review by experts prior to publication, and information on procedures for updating the guideline; and (iii) guideline development outcomes: number of recommendations with class of recommendation I, IIa, IIb, and III, and LOE A, B, and C.

### Rigour assessment

Rigour of guideline development was assessed using the eight items from the third domain of the AGREE-II instrument (see Supplementary material online, *Table S2*).^1,10^ Each was assessed independently by two reviewers (D.A.G. and R.P.). In all cases, supplementary material and societal websites were searched for additional information on the development process. Full agreement for final judgement was required and disagreements were resolved via discussion or through the involvement of a third referee, blinded for the other reviewer's assessment (M.K.).

For assessing the degree of traceability of published evidence in the produced recommendations, we have also applied the model previously suggested by Trevisiol *et al*.^4^ Guidelines were therefore classified as documents without declaration of a literature analysis or systematic review (noLA and noSR, respectively) or as stating the use of systematic search methods [SRnoDT (no details on systematic search methods), SRinsDT (insufficient methods reporting), and SR (systematic review that complies with pre-specified quality criteria)].

### Data synthesis and analysis

The main outcomes for this systematic review were (i) rate of each class of recommendation and LOE and (ii) AGREE-II tool domain 3 scores (individually and average of the eight items) and (iii) citations adjusted by year of publication (citation/guideline/year).

Categorical variables were described as numbers and frequencies, considering the unit of analysis being the individual guidance document. Continuous variables were presented as mean with SD or medians and interquartile range (IQR), as appropriate.

The following planned sensitivity/subgroup analyses were performed: (i) ESC guidelines vs. ACC/AHA guidelines and (ii) according to cardiovascular subspecialty: arrhythmias and electrophysiology, coronary artery disease, heart failure and myocardial disease, congenital and heart valve disease, cardiovascular prevention, general cardiology, and vascular medicine.^8^

## Results

### Guideline's selection and characteristics

A total of 64 documents were identified through ESC and ACC/AHA online library searching. An additional 187 records were identified from manual PubMED searches. Following screening, 89 guidelines were selected for full-text review, and an additional 13 were excluded due to not presenting summarized recommendation tables organized by both class of recommendation and LOE, or due to lack of representativeness of the body of evidence for an entire domain (i.e. <20 recommendations) (see Supplementary material online, *Figure S1*). Overall, 47 ESC (62%) and 29 ACC/AHA (38%) guidelines were included in the analysis.

**Figure 1:**
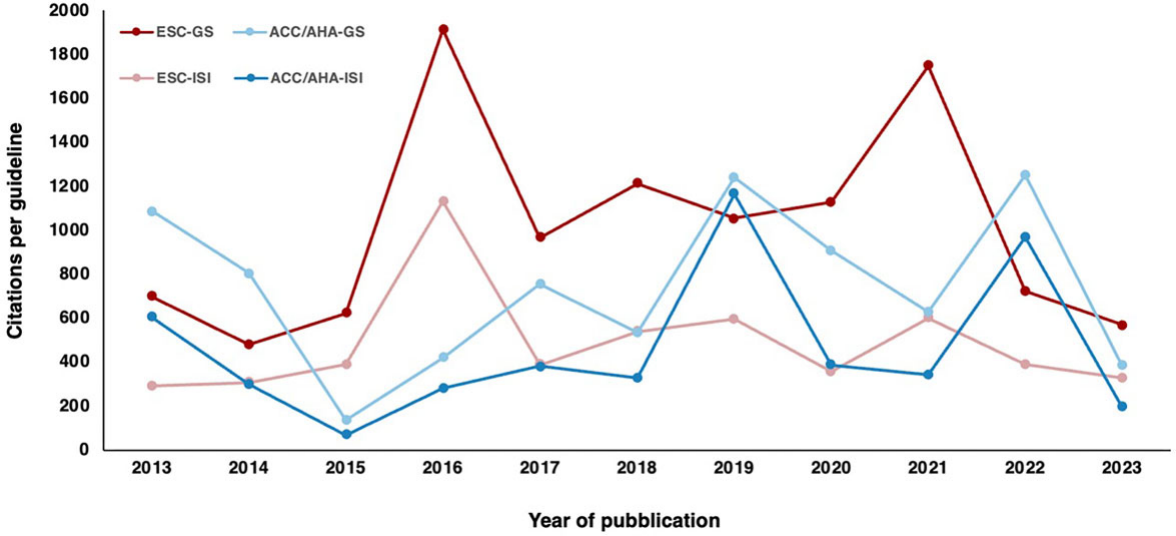
Yearly-adjusted citations per individual guideline published by the ESC and the ACC/AHA from 2012 to 2023. *Guidelines published in 2024 were not included in this analysis. ACC/AHA, American College of Cardiology/American Heart Association; ESC, European Society of Cardiology; GS, Google Scholar; ISI, ISI Web of Science.

The main characteristics of each guideline are detailed in *Table 1* and Supplementary material online, *Tables S3* and *S4*. Of the included guidelines, 44 (58%) were developed in collaboration with other international scientific societies and associations and each document was simultaneously published in a median of 2 (IQR 1–3) journals. A total of 38 (IQR 25–53) versions per guideline and translations in different languages were available to the scientific community at the time of data collection. The average number of yearly-adjusted citations in indexed journals per guideline was 344 and 681, according to ISI Web of Science and Google Scholar, respectively (the least cited document had 45 and 135 annual citations, respectively; and the most cited document had 1834 and 3490 citations per year) (*Figure 1*).

**Table 1:** Characteristics of the included guidelines

	All (*N* = 76)	ESC (*N* = 47)	ACC/AHA (*N* = 29)
Year of publication (*n*, %)			
2013–2016	23 (30.2%)	14 (29.8%)	9 (31.0%)
2017–2020	29 (38.2%)	17 (36.2%)	12 (41.4%)
2021–2024	24 (31.6%)	16 (34.0%)	8 (27.6%)
Guidelines by cardiovascular domain (*n*, %)			
Arrhythmias and electrophysiology	13 (17.1%)	7 (14.9%)	6 (20.7%)
Cardiovascular prevention	13 (17.1%)	9 (19.1%)	4 (13.8%)
Coronary artery disease	15 (19.7%)	10 (21.3%)	5 (17.2%)
Heart failure and myocardial disease	9 (11.8%)	4 (8.5%)	5 (17.2%)
Congenital and valvular heart disease	10 (19.7%)	7 (14.9%)	3 (10.3%)
General cardiology	9 (11.8%)	6 (12.8%)	3 (10.3%)
Vascular medicine	7 (9.2%)	4 (8.5%)	3 (10.3%)
Published journals (median [IQR])	2 (1–3)	1 (1–2)	3 (2–3)
Number of versions per guideline^a^ (median [IQR])	38 (25–53)	37 (25–51)	43 (30–55)
Citations adjusted by year of publication (citation/guideline/year)^a^ (median [IQR])			
Google Scholar	681 (431–1360)	689 (467–1410)	629 (418–993)
ISI Web of Science	344 (195–634)	354 (197–717)	320 (187–587)
AGREE II domain 3 (mean ± SD)			
Point 7 (systematic search methods)	3.0 ± 2.5	1.2 ± 0.9	5.9 ± 0.9
Point 8 (criteria for selecting evidence)	2.0 ± 1.7	1.1 ± 0.9	3.4 ± 1.9
Point 9 (description of strengths and limitations of the evidence)	3.4 ± 1.7	2.4 ± 1.1	5.0 ± 1.2
Point 10 (methods for formulating recommendations)	4.0 ± 0.1	4.0 ± 0.0	4.0 ± 0.2
Point 11 (risk–benefit considerations)	6.3 ± 0.5	6.3 ± 0.5	6.3 ± 0.5
Point 12 (link between recommendations and body of evidence)	6.3 ± 0.5	6.0 ± 0.0	6.8 ± 0.4
Point 13 (external review of the guideline)	2.0 ± 0.2	2.0 ± 0.1	2.0 ± 0.2
Point 14 (guideline updating procedures)	1.6 ± 1.1	1.0 ± 0.0	2.7 ± 1.1
Overall	28.6 ± 6.4	24.0 ± 2.6	36.2 ± 3.4

^a^Guidelines published in 2024 were not included in this analysis.

### Strength of recommendations and rigour of methodological development

**Figure 2:**
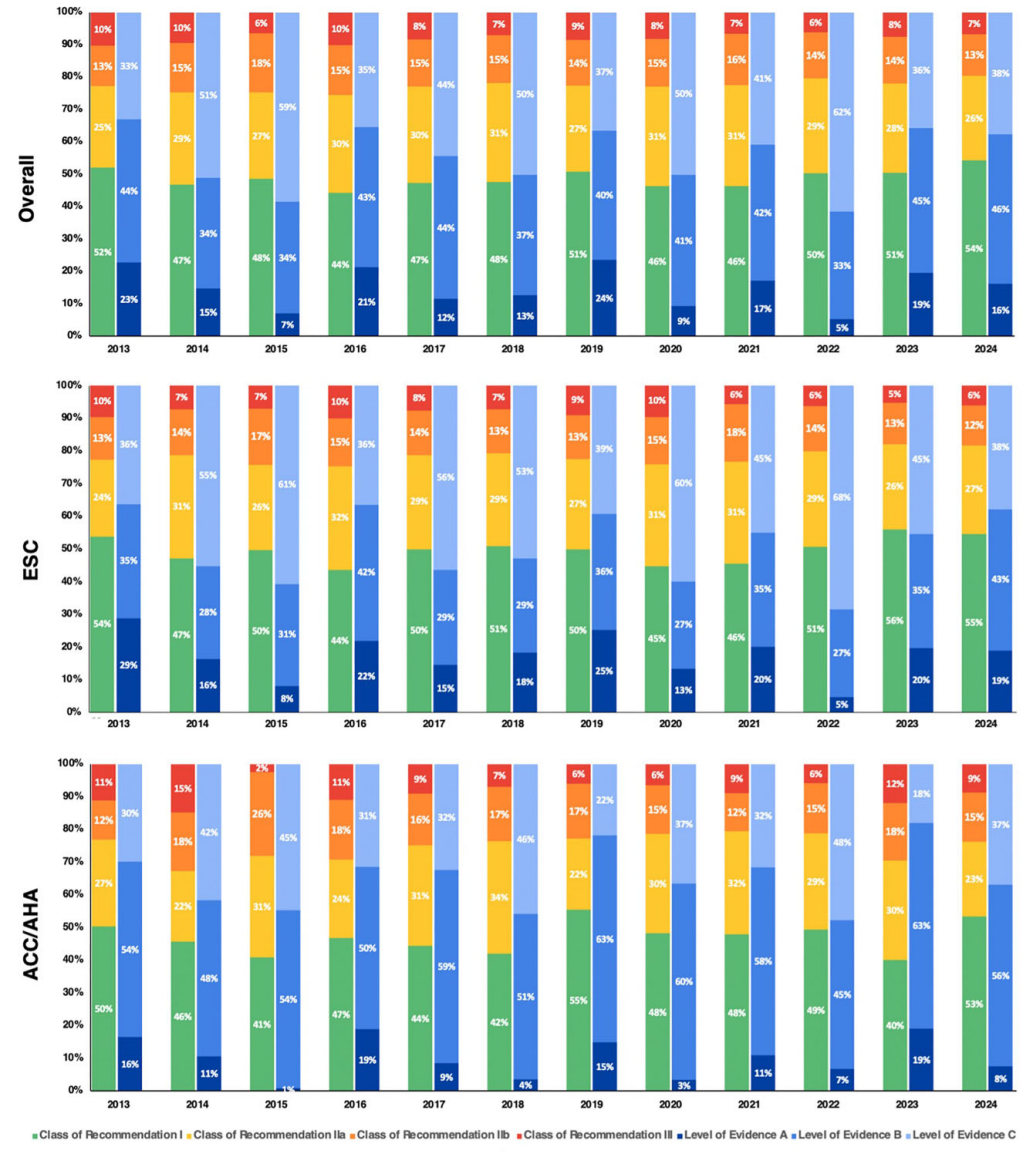
Proportion of each class of recommendation and level of evidence of the included guidelines from 2013 to 2024 (upper panel—all guidelines; middle panel—ESC guidelines; and lower panel—ACC/AHA guidelines). ACC/AHA, American College of Cardiology/American Heart Association; ESC, European Society of Cardiology.

Across the 76 included guidelines, 5338 recommendations (48.9%) were classified as class of recommendation I, 3132 (28.7%) as Class IIa, 1606 (14.7%) as Class IIb, and 847 (7.8%) as Class III. Regarding the scientific support for each recommendation, 1535 (14.1%) were classified as LOE A, while most (46.0%, 5023) were categorized as LOE C (expert opinion) (*Figure 2* and see Supplementary material online, *Table S3*). During the 12-year period the proportion of recommendations with class of recommendation I or LOE A remained consistent (*Figure 2*). Of note, in the 2024 guidelines, a minority of recommendations (16.2%) were informed by solid evidence from randomized trials.

**Figure 3:**
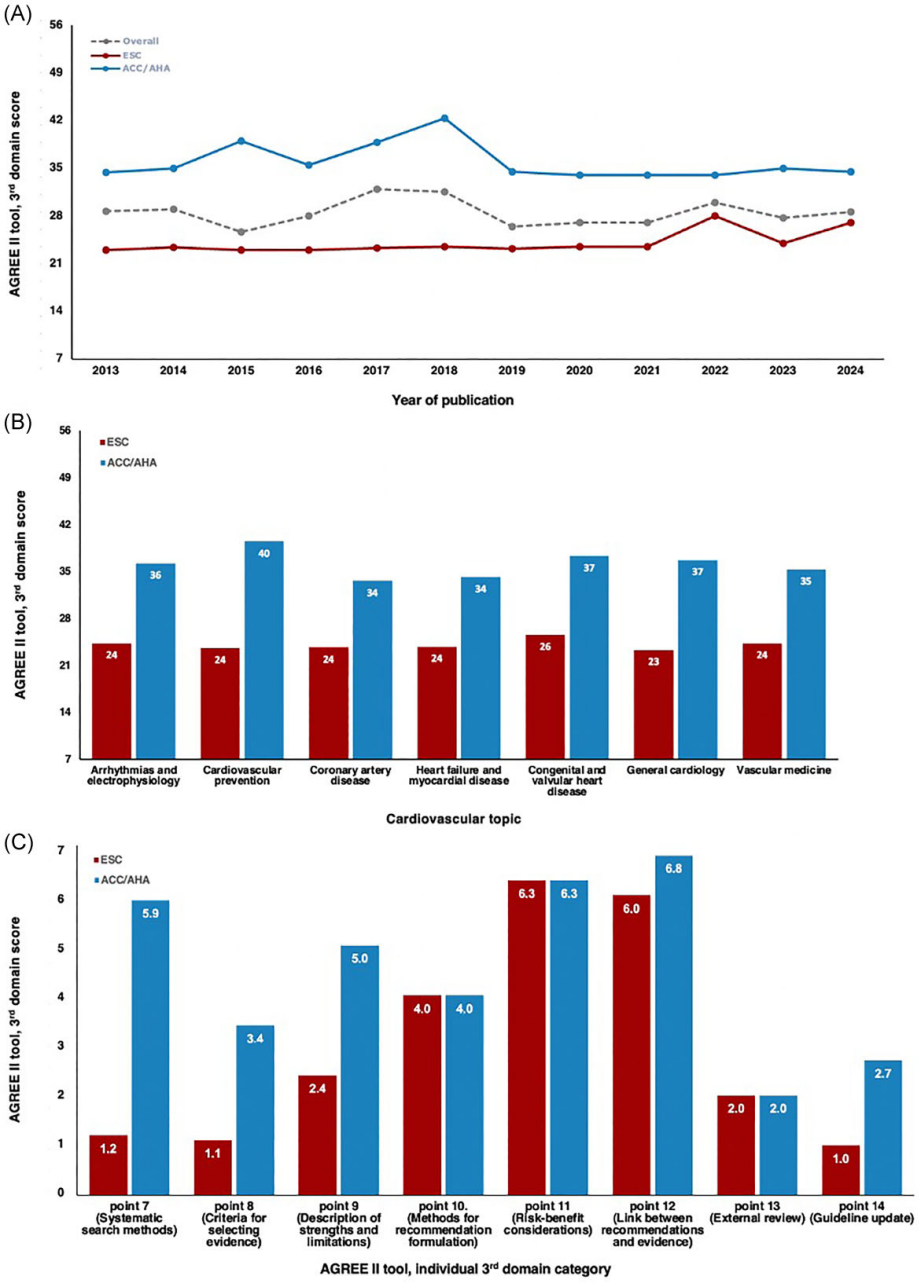
(*A*) Evolution of AGREE II tool third domain scores from 2013 to 2024. (*B*) AGREE II tool third domain scores according to cardiovascular topic. (*C*) Overall individual scores on each AGREE II tool third domain category.

Regarding the rigour of methodological development, the overall AGREE II domain 3 score was 29 ± 6 (out of 56 possible points). The third domain categories in which the guidelines scored lowest were the use of systematic methods for evidence search (point 7), the establishment of criteria for selecting evidence (point 8), the traceability of the external review (point 13), and the establishment of a procedure for updating the guideline (point 14). Interestingly, there were no significant variations regarding rigour of methodological development over the last years (*Figure 3**A* and see Supplementary material online, *Table S4*).

### Comparison between ESC and ACC/AHA guidelines

Overall, 2260 (65.1%) of the ACC/AHA guideline recommendations were evidence-based (LOE A and B), while 1261 (34.9%) derived from expert consensus alone. On the contrary, most of the ESC recommendations were based on expert opinion [3762 (50.8%)] (*Figure 2*).

The rigour of guideline development also varied significantly across the Atlantic. The mean overall score of the AGREE II third domain of ACC/AHA guidelines was 36.2 ± 3.4 vs. 24.0 ± 2.6 of the ESC recommendations. The major differences refer to the use of systematic methods to search for evidence (point 7), the use of pre-defined inclusion and exclusion criteria for selecting the evidence (point 8), and the use of formal assessment of strengths and limitations of the body of evidence, including evaluation for bias (point 9) (see Supplementary material online, *Table S4*). Nonetheless, two interesting aspects should be noted. Firstly, the rigour of development of ACC/AHA guidelines appeared to be improving until 2018, with the ones on bradycardia and cardiac conduction delays^11^ and on adult congenital heart disease^12^ scoring as high as 43 points on the third domain (see Supplementary material online, *Table S4*). Notably, after 2018, a decrease in the overall score was observed, which has remained fairly stable ever since (*Figure 3*). Secondly, when compared with the remaining European recommendations, the 2022 ESC guidelines showed a higher AGREE II third domain score. This was mainly driven by the pulmonary hypertension guideline, which was developed in collaboration with the European Respiratory Society.^13^ As opposed to the remaining, in this document, a methodology of systematic review, inclusion and exclusion criteria, and formal assessment of strengths and limitations was clearly stated. Although some improvement in the ESC 2024 guidelines was observed in association with the publication of detailed evidence tables for each recommendation, the lack of a systematic review of evidence remains one of the main limitations (*Figure 3B* and *C*).

In a total of 9 guidelines (12%; all from ACC/AHA), commissioned systematic reviews were conducted to address clinical questions identified by the expert writing panel.^11,12,14–20^ The classification of the degree of traceability of published evidence in the generated recommendations is detailed in Supplementary material online, *Table S5*. Briefly, in 45 (96%) of the ESC guidelines there was no declaration of a systematic search of evidence, whereas 27 (93%) of those developed by the ACC/AHA state that a systematic search was conducted and provide additional details regarding its methods.

### Comparison by cardiovascular domain

**Figure 4:**
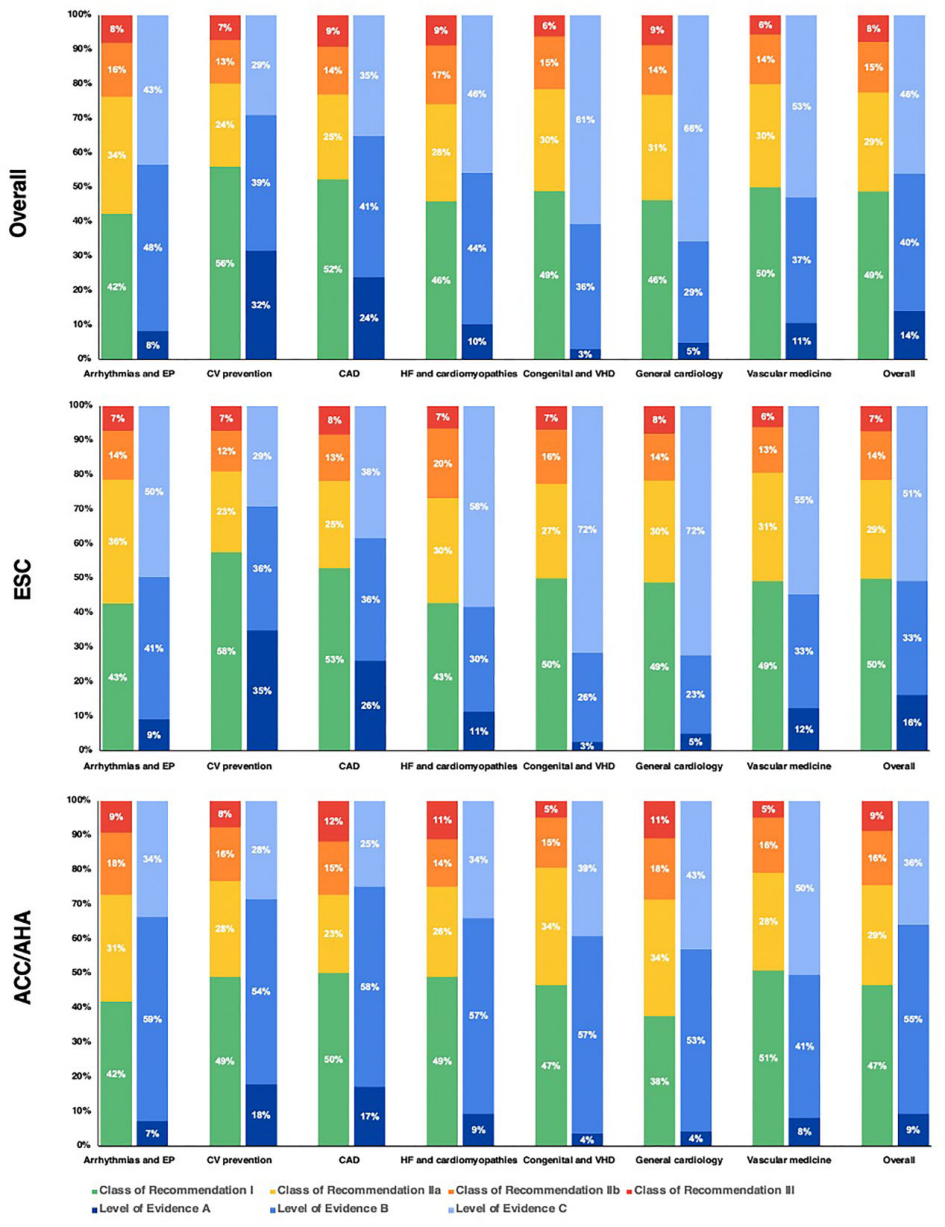
Proportion of each class of recommendation and level of evidence according to cardiovascular domain (upper panel—all guidelines; middle panel—ESC guidelines; and lower panel—ACC/AHA guidelines). ACC/AHA, American College of Cardiology/American Heart Association; ESC, European Society of Cardiology.

The rate of classes of recommendation and LOE stratified by main guideline domain can be found in *Figure 4*. Across both ESC and ACC/AHA guidelines, the documents on arrhythmias and electrophysiology and those on congenital and valvular heart disease and general cardiology showed the lowest proportion of LOE A recommendations (ranging 3–9%). The rate of Class I recommendations was comparable across the different cardiovascular domains, except for arrhythmia and electrophysiology where it was lower.

The AGREE II third domain scores according to cardiovascular domain and society are depicted in *Figure 3**B* and in Supplementary material online, *Table S6*.

## Discussion

In this systematic review, we aimed to assess the overall recommendations’ strength and the rigour of development of clinical practice guidelines from the main European and American scientific cardiovascular societies. Our main findings can be described as follows:

- Clinical guidelines from the main European and American scientific cardiovascular societies have an important impact and influence as measured by their annual adjusted citation count.
- Despite years of scientific progress and of being exceedingly popular amongst the scientific community, less than 15% of recommendations are based on solid evidence from randomized trials or meta-analyses.
- There are significant limitations in the methodological rigour of guidelines’ development, particularly regarding the performance of a systematic literature review and assessment of the body of evidence. This is demonstrated by an overall score on the AGREE II third domain of 29 (out of a possible maximum of 56).
- Compared with ACC/AHA guidelines, ESC recommendations are less likely to be evidence-based and show far greater deficiencies regarding the methodology of development.

Clinical practice guidelines are commonly defined as systematically developed documents to assist in decision-making for a specific health circumstance and have become an increasingly popular tool for synthesis of information over the past 20 years.^2,21^ The main goals of guidelines are to improve quality of care and to identify areas of future research. ESC and ACC/AHA guidelines are amongst the most important cardiovascular disease guidelines in medicine, and the distinctively high number of versions, translations, and citations underscore their popularity among the physicians.^6–8^ In fact, previous studies have assessed the remarkable real-world applicability of such guidance while others have summarized strategies for improving implementation.^22,23^

### Strength of recommendations

Over a 12-year period, we observed that the proportion of class of recommendation I remained stable at approximately 50%. A particularly striking finding is that, despite the widespread use and popularity of these guidelines, nearly half of the recommendations were based primarily on expert opinion rather than robust scientific evidence. This is even more notable considering that the overall proportion of LOE A (14%) and C (46%) recommendations shows a fairly stable trend over time. In other words, this suggests that, despite the growing body of scientific evidence, the number of recommendations supported by high-quality research has not significantly increased.

Taken together, these findings indicate that efforts in recent decades to support and enhance RCTs have not yet led to more evidence-based recommendations.^8^ Without robust and well-conducted RCT-based evidence, the link between clinical practice and cardiovascular outcomes may be less reliable.^8^ Even though organizations like the World Health Organization and the National Institute for Health and Care Excellence develop efforts to provide evidence based on RCTs, in some cases this may not be possible.^24^ Hence, providing recommendations solely from randomized evidence may be an elusive goal. Although this may seem paradoxical at first sight, some of the LOE C recommendations ensure that guidelines remain centred in improving patient care.^24^ For instance, it is highly unlikely that an RCT will ever be conducted to confirm that all patients presenting with ventricular arrhythmia should be investigated for reversible causes. On the other hand, large registries like SWEDEHEART^25^ and nationwide datasets from the UK,^26^ Denmark,^27^ Finland,^28^ and the USA^29^ have been widely used to advance knowledge of cardiovascular disease. Better communication between guideline committees and researchers, with requests for data outputs and manuscripts addressing specific questions and recommendations under consideration, may help generate evidence support to grant LOE B instead LOE C to many recommendations. To exemplify, the recent 2024 ESC atrial fibrillation guidelines recommend the adoption of the CHA_2_DS_2_VA score supported by a LOE C,^30^ but a recent analysis of the UK nationwide dataset could have provided a LOE B or higher.^28^

### Methodological rigour assessment

Methodologically, it is well-established that clinical practice guidelines must clearly demonstrate the use of a systematic search strategy and the execution of systematic review steps.^2^ The updated AGREE II tool, introduced in 2010 by Brouwers *et al*.^31^ and subsequently validated,^10^ provides an objective framework for measuring and comparing the methodological rigour of clinical guidelines. Our study shows significant deficiencies regarding scientific rigour of development, underscored by an AGREE II third domain score of 29. Strikingly, over the years, and despite clear recommendations from the National Academy of Medicine^2^ on guideline development, overall rigour did not seem to improve. Altogether, these findings are in line with those reported by Alonso-Coello *et al*.^32^: in a systematic review of clinical practice guidelines across a wide range of healthcare domains over a span of almost 30 years, the methods quality scores remained low to moderate. Padjas and colleagues^33^ presented similar results when analysing the methodological rigour and reporting of clinical practice guidelines in patients with allergic rhinitis. In our study, the methodological quality varied significantly according to the developer organization. Compared with ACC/AHA, ESC guidelines were far less likely to report a systematic review during the development process (AGREE II third domain 36 vs. 24, respectively).

Clinical practice guidelines are a major source of information for clinicians worldwide, and their main goal is to improve patients’ care. Accordingly, they should follow traceable and reproducible methods of systematic review and should perform a formal and critical appraisal of strengths and limitations of the body of evidence.^3^ Otherwise, selective reporting of evidence during the elaboration recommendations may ultimately jeopardize outcomes. The same principles could be applied to RCTs. For example, it is not currently acceptable to start recruitment before the registration and publication of the methodology and endpoints of interest.

Understanding the need for better-quality and reproducible guidelines, several national and international organizations have adopted stringent criteria for using evidence to guide recommendations. In the guidelines developed by the World Health Organization, the expert committee identifies relevant search questions, which are then critically assessed by means of commissioned systematic reviews and meta-analyses published in peer-review journals.^34–36^ In the field of cardiovascular disease, the 2018 Australian Clinical Guidelines on atrial fibrillation is another example in which state-of-the-art methods for evidence synthesis were employed.^37^ In both cases, all available evidence is formally assessed for risk of bias using the Grading of Recommendations, Assessment, Development, and Evaluation (GRADE) methodology before issuing a recommendation.^38^

### Limitations and strengths

The results of this systematic review should be interpreted considering several limitations. Firstly, we considered all published guidelines from the ACC/AHA and the ESC since 2013, including those that have now been replaced by updated documents. The rationale was to create a full picture of the evolution of the recommendations, evidence, and development methodology over the years. We have also excluded guidelines and/or focused updates on specific chapters with fewer than 20 recommendations. Although this was an arbitrary cut-off (excluded guidelines had as few as 9 ± 4 recommendations—Supplementary material online, *Table S7*), it was motivated by an effort to avoid including documents that might not be representative of the entire body of evidence on a given domain. Finally, we appreciate that a low score on the AGREE II tool does not mean that a guideline is not useful in clinical practice and does not assess the clinical importance and appropriateness of recommendations. It does mean, however, that appropriate review and reporting may not have been followed.

To our best knowledge, this is the first study systematically assessing the quality of the main cardiovascular societies’ guidelines over a period that spans for over a decade. We have demonstrated that, for most of the ACC/AHA and ESC guidelines, there is significant room for improvement in the rigour of their development and quality of their reporting, which will likely improve their impact for patient care. Given the importance of these documents for clinical practice and for improving patients’ outcomes, we do believe that the development process should follow strict reporting, reproducible and transparent methods, including a peer-reviewed systematic literature review to inform recommendations. We hope these results will urge guideline authors and societies to provide higher-quality recommendations, ultimately offering clinicians the best information to make informed decisions about individual patient care.

## Conclusions

Despite their widespread influence and popularity, clinical practice guidelines from the main European and American cardiovascular societies exhibit considerable shortcomings in methodological rigour, with no notable improvements over the last 12 years. The ESC recommendations rely more often on expert opinion and demonstrate greater methodological deficiencies compared with the ACC/AHA guidance. All efforts should be made to improve the rigour and transparency of future guidelines development.

## Supplementary Material

qcae113_Supplemental_File

## Data availability

The data supporting the findings of this study are available within the article and its supplementary materials.

## References

[1] Brouwers MC, Kerkvliet K, Spithoff K. AGREE Next Steps Consortium. The AGREE Reporting Checklist: a tool to improve reporting of clinical practice guidelines. BMJ 2016;352:i1152. 26957104 10.1136/bmj.i1152 PMC5118873

[2] Agency for Healthcare Research and Quality. NGC and NQMC Inclusion Criteria. Rockville: Agency for Healthcare Research and Quality; 2018. https://www.ahrq.gov/gam/summaries/inclusion-criteria/index.html (accessed 3 September 2024).

[3] Brouwers MC, Kho ME, Browman GP, Burgers JS, Cluzeau F, Feder G et al. AGREE Next Steps Consortium. AGREE II: advancing guideline development, reporting and evaluation in health care. CMAJ 2010;182:E839–E842. 20603348 10.1503/cmaj.090449 PMC3001530

[4] Trevisiol C, Cinquini M, Fabricio ASC, Gion M, Rutjes AWS. Insufficient uptake of systematic search methods in oncological clinical practice guideline: a systematic review. BMC Med Res Methodol 2019;19:180. 31429714 10.1186/s12874-019-0818-5 PMC6702747

[5] Grilli R, Magrini N, Penna A, Mura G, Liberati A. Practice guidelines developed by specialty societies: the need for a critical appraisal. Lancet North Am Ed 2000;355:103–106. 10.1016/S0140-6736(99)02171-6 10675167

[6] Tantawy M, Marwan M, Hussien S, Tamara A, Mosaad S. The scale of scientific evidence behind the current ESC clinical guidelines. Int J Cardiol Heart Vasc 2023;45:101175. 37070121 10.1016/j.ijcha.2023.101175 PMC10105211

[7] Tricoci P, Allen JM, Kramer JM, Califf RM, Smith SC Jr. Scientific evidence underlying the ACC/AHA clinical practice guidelines. JAMA 2009;301:831–841. 19244190 10.1001/jama.2009.205

[8] Fanaroff AC, Califf RM, Windecker S, Jr SSC, Lopes RD. Levels of evidence supporting American College of Cardiology/American Heart Association and European Society of Cardiology Guidelines, 2008–2018. JAMA 2019;321:1069–1080. 30874755 10.1001/jama.2019.1122 PMC6439920

[9] Page MJ, McKenzie JE, Bossuyt PM, Boutron I, Hoffmann TC, Mulrow CD et al. The PRISMA 2020 statement: an updated guideline for reporting systematic reviews. BMJ 2021;372:n71. 33782057 10.1136/bmj.n71 PMC8005924

[10] Brouwers MC, Kho ME, Browman GP, Burgers JS, Cluzeau F, Feder G et al. Development of the AGREE II, part 2: assessment of validity of items and tools to support application. CMAJ 2010;182:E472–E478. 20513779 10.1503/cmaj.091716 PMC2900368

[11] Kusumoto FM, Schoenfeld MH, Barrett C, Edgerton JR, Ellenbogen KA, Gold MR et al. 2018 ACC/AHA/HRS guideline on the evaluation and management of patients with bradycardia and cardiac conduction delay: a report of the American College of Cardiology/American Heart Association Task Force on Clinical Practice Guidelines and the Heart Rhythm Society. Circulation 2019;140:e382–e482. 30586772 10.1161/CIR.0000000000000628

[12] Stout KK, Daniels CJ, Aboulhosn JA, Bozkurt B, Broberg CS, Colman JM et al. 2018 AHA/ACC Guideline for the management of adults with congenital Heart disease: a report of the American College of Cardiology/American Heart Association Task Force on clinical Practice Guidelines. Circulation 2019;139:e698–e800. 30586767 10.1161/CIR.0000000000000603

[13] Humbert M, Kovacs G, Hoeper MM, Badagliacca R, Berger RMF, Brida M et al.; ESC/ERS Scientific Document Group. 2022 ESC/ERS Guidelines for the diagnosis and treatment of pulmonary hypertension. Eur Respir J 2023;61:2200879. 36028254 10.1183/13993003.00879-2022

[14] Page RL, Joglar JA, Caldwell MA, Calkins H, Conti JB, Deal BJ et al.; Evidence Review Committee Chair. 2015 ACC/AHA/HRS guideline for the management of adult patients with supraventricular tachycardia: executive summary: a report of the American College of Cardiology/American Heart Association Task Force on Clinical Practice Guidelines and the Heart Rhythm Society. Circulation 2016;133:e471–e505. 26399662 10.1161/CIR.0000000000000310

[15] Fleisher LA, Fleischmann KE, Auerbach AD, Barnason SA, Beckman JA, Bozkurt B et al. 2014 ACC/AHA guideline on perioperative cardiovascular evaluation and management of patients undergoing noncardiac surgery: a report of the American College of Cardiology/American Heart Association Task Force on Practice Guidelines. Circulation 2014;130:e278–e333. 25085961 10.1161/CIR.0000000000000106

[16] Levine GN, Bates ER, Bittl JA, Brindis RG, Fihn SD, Fleisher LA et al. 2016 ACC/AHA guideline focused update on duration of dual antiplatelet therapy in patients with coronary artery disease: a report of the American College of Cardiology/American Heart Association Task Force on clinical Practice guidelines. J Am Coll Cardiol 2016;68:1082–1115. 27036918 10.1016/j.jacc.2016.03.513

[17] Shen WK, Sheldon RS, Benditt DG, Cohen MI, Forman DE, Goldberger ZD et al. 2017 ACC/AHA/HRS Guideline for the evaluation and management of patients with syncope: a report of the American College of Cardiology/American Heart Association Task Force on Clinical Practice Guidelines and the Heart Rhythm Society. Circulation 2017;136:e60–e122. 28280231 10.1161/CIR.0000000000000499

[18] Al-Khatib SM, Stevenson WG, Ackerman MJ, Bryant WJ, Callans DJ, Curtis AB et al. 2017 AHA/ACC/HRS guideline for management of patients with ventricular arrhythmias and the prevention of sudden cardiac death: a report of the American College of Cardiology/American Heart Association Task Force on Clinical Practice Guidelines and the Heart Rhythm Society. J Am Coll Cardiol 2018;72:e91–e220. 29097296 10.1016/j.jacc.2017.10.054

[19] Whelton PK, Carey RM, Aronow WS, Casey DE Jr, Collins KJ, Dennison Himmelfarb C et al. 2017 ACC/AHA/AAPA/ABC/ACPM/AGS/APhA/ASH/ASPC/NMA/PCNA Guideline for the prevention, detection, evaluation, and management of high blood pressure in adults: a report of the American College of Cardiology/American Heart Association Task Force on Clinical Practice guidelines. Circulation 2018;138:e484–e594. 30354654 10.1161/CIR.0000000000000596

[20] Grundy SM, Stone NJ, Bailey AL, Beam C, Birtcher KK, Blumenthal RS et al. 2018 AHA/ACC/AACVPR/AAPA/ABC/ACPM/ADA/AGS/APhA/ASPC/NLA/PCNA Guideline on the Management of blood Cholesterol: a report of the American College of Cardiology/American Heart Association Task Force on Clinical Practice Guidelines. J Am Coll Cardiol 2019;73:e285–e350. 30423393 10.1016/j.jacc.2018.11.003

[21] Klein WW . Current and future relevance of guidelines. Heart 2002;87:497–500. 12010924 10.1136/heart.87.6.497 PMC1767136

[22] Chan WV, Pearson TA, Bennett GC, Cushman WC, Gaziano TA, Gorman PN et al. ACC/AHA special Report: clinical practice guideline implementation strategies: a summary of systematic reviews by the NHLBI Implementation Science Work Group: a report of the American College of Cardiology/American Heart Association Task Force on Clinical Practice Guidelines. J Am Coll Cardiol 2017;69:1076–1092. 28132746 10.1016/j.jacc.2016.11.004

[23] van Dijk WB, Schuit E, van der Graaf R, Groenwold RHH, Laurijssen S, Casadei B et al. Applicability of European Society of Cardiology guidelines according to gross national income. Eur Heart J 2023;44:598–607. 36396400 10.1093/eurheartj/ehac606 PMC9925274

[24] Bonow RO, Braunwald E. The evidence supporting cardiovascular guidelines: is there evidence of progress in the last decade? JAMA 2019;321:1053–1054. 30874738 10.1001/jama.2019.2018

[25] Hofmann R, James SK, Jernberg T, Lindahl B, Erlinge D, Witt N et al. Oxygen therapy in suspected acute myocardial infarction. N Engl J Med 2017;377:1240–1249. 28844200 10.1056/NEJMoa1706222

[26] Chung SC, Providencia R, Sofat R. Association between angiotensin blockade and incidence of influenza in the United Kingdom. N Engl J Med 2020;383:397–400. 32383830 10.1056/NEJMc2005396 PMC7233186

[27] Olesen JB, Lip GYH, Kamper AL, Hommel K, Køber L, Lane DA et al. Stroke and bleeding in atrial fibrillation with chronic kidney disease. N Engl J Med 2012;367:625–635. 22894575 10.1056/NEJMoa1105594

[28] Teppo K, Lip GYH, Airaksinen KEJ, Halminen O, Haukka J, Putaala J et al. Comparing CHA2DS2-VA and CHA2DS2-VASc scores for stroke risk stratification in patients with atrial fibrillation: a temporal trends analysis from the retrospective Finnish AntiCoagulation in Atrial Fibrillation (FinACAF) cohort. Lancet Reg Health Eur 2024;43:100967. 39171253 10.1016/j.lanepe.2024.100967 PMC11337097

[29] McCarthy CP, Kolte D, Kennedy KF, Vaduganathan M, Wasfy JH, Januzzi JL. Patient characteristics and clinical outcomes of type 1 versus type 2 myocardial infarction. J Am Coll Cardiol 2021;77:848–857. 33602466 10.1016/j.jacc.2020.12.034

[30] Van Gelder IC, Rienstra M, Bunting KV, Casado-Arroyo R, Caso V, Crijns HJGM et al.; ESC Scientific Document Group. 2024 ESC guidelines for the management of atrial fibrillation developed in collaboration with the European Association for Cardio-Thoracic Surgery (EACTS). Eur Heart J 2024;45:3314–3414. 39210723 10.1093/eurheartj/ehae176

[31] Brouwers MC, Kho ME, Browman GP, Burgers JS, Cluzeau F, Feder G et al. Development of the AGREE II, part 1: performance, usefulness and areas for improvement. CMAJ 2010;182:1045–1052. 20513780 10.1503/cmaj.091714 PMC2900328

[32] Alonso-Coello P, Irfan A, Solà I, Gich I, Delgado-Noguera M, Rigau D et al. The quality of clinical practice guidelines over the last two decades: a systematic review of guideline appraisal studies. Qual Saf Health Care 2010;19:e58. 21127089 10.1136/qshc.2010.042077

[33] Padjas A, Kehar R, Aleem S, Mejza F, Bousquet J, Schünemann HJ et al. Methodological rigor and reporting of clinical practice guidelines in patients with allergic rhinitis: quGAR study. J Allergy Clin Immunol 2014;133:777–783.e4. 24139606 10.1016/j.jaci.2013.08.029

[34] Guidelines Review Committee . WHO handbook for guidelines development. Criteria for use of evidence to inform recommendations in World Health Organization guidelines. 2023 Nov. https://www.who.int/publications/i/item/WHO-SCI-QNS-MST-2023.1 (accessed 3 September 2024).

[35] Providência R, Aali G, Zhu F, Katairo T, Ahmad M, Bray JJH et al. Handheld echocardiography for the screening and diagnosis of rheumatic heart disease: a systematic review to inform WHO guidelines. Lancet Glob Health 2024;12:e983–e994. 38762298 10.1016/S2214-109X(24)00127-X

[36] Providencia R, Aali G, Zhu F, Leas BF, Orrell R, Ahmad M et al. Penicillin allergy testing and delabeling for patients who are prescribed Penicillin: a systematic review for a World Health Organization guideline. Clinic Rev Allerg Immunol 2024;66:223–240. 10.1007/s12016-024-08988-2 PMC11193836 38696031

[37] Brieger D, Amerena J, Attia J, Bajorek B, Chan KH, Connell C et al. National Heart Foundation of Australia and the Cardiac Society of Australia and New Zealand: Australian Clinical Guidelines for the Diagnosis and Management of atrial Fibrillation 2018. Heart Lung Circ 2018;27:1209–1266. 30077228 10.1016/j.hlc.2018.06.1043

[38] Guyatt GH, Oxman AD, Vist GE, Kunz R, Falck-Ytter Y, Alonso-Coello P et al. GRADE Working Group. GRADE: an emerging consensus on rating quality of evidence and strength of recommendations. BMJ 2008;336:924–926. 18436948 10.1136/bmj.39489.470347.AD PMC2335261

