# Supplementary material for: Rigour of development of European Society of Cardiology, American College of Cardiology and American Heart Association guidelines over a 12-year period (2013–2024): a systematic review of guidelines
Source: Eur Heart J Qual Care Clin Outcomes. 2025 Jan 20;11(6):875–85. doi: 10.1093/ehjqcco/qcae113 (PMC12445687; doi:10.1093/ehjqcco/qcae113)
Supplement: qcae113_Supplemental_File [file qcae113_supplemental_file.docx]

**SUPPLEMENTARY MATERIAL**

**Table of contents**

Supplementary Table 1………………………………………………………………………………………………………………………………2

Supplementary Table 2………………………………………………………………………………………………………………………………3

Supplementary Table 3………………………………………………………………………………………………………………………………6

Supplementary Table 4…………………………………………………………………………………………………………………………...…18

References for Supplementary Tables 3 and 4………………………………………………………………….………………………………...…25

Supplementary Table 5……………………………………………………………………………………………………………………………...34

Supplementary Table 6……………………………………………………………………………………………………………………………...35

Supplementary Table 7……………………………………………………………………………………………………………………………...36

References for Supplementary Table 7……………………………………………………………………………………………………………...36

Supplementary Figure 1...…………………………………………………………………………………………………………………………...37

PRISMA checklist…………………………………………………………………………………………………………………………………..38

**Supplementary Table 1** – Classes of recommendation and levels of evidence.

| **Class of Recommendation** | |
| --- | --- |
| **I** | Evidence and/or general agreement that a given treatment or procedure is beneficial and effective. |
| **IIa** | Weight of evidence/opinion is in favour of efficacy. |
| **IIb** | Usefulness is less well established by evidence/opinion |
| **III** | Evidence or general agreement that the given treatment or procedure is not effective and may be harmful. |
| **Level of Evidence (LOE)** | |
| **LOE A** | Data derived from multiple randomised clinical trials or meta-analyses. |
| **LOE B** | Data derived from a single randomised clinical trial or large non-randomised studies. |
| **LOE C** | Consensus of experts and/or small studies, retrospective studies, registries. |

**Supplementary Table 2** – Rigour of guidelines scoring system using AGREE II tool 3rd-domain. Each item is scored from 1 (lowest rigour of development) to 7 (highest rigour). Scoring points are added to 1 up to a maximum of 7 points per item.

| **AGREE II - 3^rd^ domain** | **Rigour assessment** | **Scoring system** |
| --- | --- | --- |
| **7.** | **Systematic methods were used to search for evidence** |  |
|  | named electronic database(s) or evidence source(s) where the search was performed | 1 point |
|  | time periods searched | 1 point |
|  | search terms used | 1 point |
|  | full search strategy included | 1 point |
|  | additional considerations:  is the item well written? Are the descriptions clear and concise?  is the item content easy to find in the guideline?  is the search relevant and appropriate to answer the health question?  is there enough information provided for anyone to replicate the search? | 2 points |
| **8.** | **The criteria for selecting the evidence are clearly described** |  |
|  | description of the inclusion criteria, including target population characteristics, study design, comparisons (if relevant), outcomes, language (if relevant), context (if relevant) | 2 points |
|  | description of the exclusion criteria (if relevant) | 2 points |
|  | additional considerations:  is the item well written? Are the descriptions clear and concise?  is the item content easy to find in the guideline?  is there a rationale given for the chosen inclusion/exclusion criteria?  do inclusion/exclusion criteria align with the health question(s)?  are there reasons to believe that relevant literature may not have been considered? | 4 points |
| **9.** | **The strengths and limitations of the body of evidence are clearly described** |  |
|  | descriptions of how the body of evidence was evaluated for bias and how it was interpreted by members of the guideline development group, including:  -study design(s) included in body of evidence  -study methodology limitations  -appropriateness/relevance of primary and secondary outcomes considered  -consistency of results across studies  -direction of results across studies  -magnitude of benefit versus magnitude of harm  -applicability to practice context | 4 points |
|  | additional considerations:  is the item well written? Are the descriptions clear and concise? Is the item content easy to find in the guideline?  are the descriptions appropriate, neutral, and unbiased? Are the descriptions complete? | 2 points |
| **10.** | **The methods for formulating the recommendations are clearly described.** |  |
|  | description of the recommendation development process (e.g., steps used in modified Delphi technique, voting procedures that were considered) | 1 point |
|  | outcomes of the recommendation development process (e.g., extent to which consensus was reached using modified Delphi technique, outcome of voting procedures) | 1 point |
|  | description of how the process influenced the recommendations (e.g., results of Delphi technique influence final recommendation, alignment with recommendations and the final vote) | 1 point |
|  | additional considerations:  is the item well written? Are the descriptions clear and concise? Is the item content easy to find in the guideline?  was a formal process used to arrive at the recommendations?  were the methods appropriate? | 3 points |
| **11.** | **The health benefits, side effects, and risks have been considered in formulating the recommendations.** |  |
|  | supporting data and report of benefits | 1 point |
|  | supporting data and report of harms/side effects/risks | 1 point |
|  | reporting of the balance/trade-off between benefits and harms/side effects/risks | 1 point |
|  | recommendations reflect considerations of both benefits and harms/side effects/risks | 1 point |
|  | additional considerations:  is the item well written? Are the descriptions clear and concise? Is the item content easy to find in the guideline?  is the discussion an integral part of the guideline development process? (i.e., taking place during recommendation formulation rather than post-formulation as an afterthought)  has the guideline development group considered the benefits and harms equally? | 2 points |
| **12.** | **There is an explicit link between the recommendations and the supporting evidence.** |  |
|  | the guideline describes how the guideline development group linked and used the evidence to inform recommendations | 1 point |
|  | each recommendation is linked to a key evidence description/paragraph and/or reference list | 1 point |
|  | recommendations linked to evidence summaries, evidence tables in the results section of the guideline | 1 point |
|  | additional considerations:  is there congruency between the evidence and recommendations?  is the link between the recommendations and supporting evidence easy to find in the guideline?  when evidence is lacking or a recommendation is informed primarily by consensus of opinion, is this clearly stated and described? | 3 points |
| **13.** | **The guideline has been externally reviewed by experts prior to its publication.** |  |
|  | purpose and intent of the external review (e.g., to improve quality, gather feedback on draft recommendations, assess applicability and feasibility, disseminate evidence) | 1 point |
|  | methods taken to undertake the external review (e.g., rating scale, open-ended questions) | 1 point |
|  | description of the external reviewers (e.g., number, type of reviewers, affiliations) | 1 point |
|  | outcomes/information gathered from the external review (e.g., summary of key findings) | 1 point |
|  | description of how the information gathered was used to inform the guideline development process and/or formation of the recommendations | 1 point |
|  | additional considerations:  is the item well written? Are the descriptions clear and concise?  is the item content easy to find in the guideline?  are the external reviewers relevant and appropriate to the scope of the guideline? Was there a rationale given for choosing the included reviewers?  how was information from the external review used by the guideline development group? | 1 point |
| **14.** | **A procedure for updating the guideline is provided** |  |
|  | a statement that the guideline will be updated | 1 point |
|  | explicit time interval or explicit criteria to guide decisions about when an update will occur | 1 point |
|  | methodology for the updating procedure is reported | 1 point |
|  | additional considerations:  is the item well written? Are the descriptions clear and concise? Is the item content easy to find in the guideline?  is there enough information provided to know when an update will occur or what criteria would trigger an update? | 3 points |

**Supplementary Table 3** – Number of citations and recommendations of the included guidelines by Class of Recommendation and Level of Evidence.

| **Guideline** | **Soc.** | **Year** | **Yearly-adjusted citations on Google Scholar** | **Yearly-adjusted citations on ISI Web of Science** | **Class of Recommendation (n, %)** | | | | **Level of Evidence (n, %)** | | | |
| --- | --- | --- | --- | --- | --- | --- | --- | --- | --- | --- | --- | --- |
|  |  |  |  |  | **I** | **IIa** | **IIb** | **III** | **A** | **B** | **C** | **Total** |
| **2013 ACCF/AHA Guideline for the Management of ST-Elevation Myocardial Infarction** | ACC/AHA | 2013 | 924 | 409 | 84 (63.6%) | 26 (19.7%) | 13 (9.8%) | 9 (6.8%) | 18 (13.6%) | 78 (59.1%) | 36 (27.3%) | 132 |
| **2013 ACCF/AHA Guideline for the Management of Heart Failure** | ACC/AHA | 2013 | 1388 | 815 | 59 (43.1%) | 41 (29.9%) | 19 (13.9%) | 18 (13.1%) | 26 (19%) | 67 (48.9%) | 44 (32.1%) | 137 |
| **2013 ACC/AHA Guideline on the Treatment of Blood Cholesterol to Reduce Atherosclerotic Cardiovascular Risk in Adults** | ACC/AHA | 2013 | 952 | 592 | 16 (34%) | 17 (36.2%) | 6 (12.8%) | 8 (17%) | 8 (17%) | 25 (53.2%) | 14 (29.8%) | 47 |
| **2013 ESC guidelines on the management of stable coronary artery disease** | ESC | 2013 | 496 | 314 | 69 (53.1%) | 26 (20%) | 22 (16.9%) | 13 (10%) | 21 (16.2%) | 44 (33.8%) | 65 (50%) | 130 |
| **2013 ESC Guidelines on diabetes, pre-diabetes, and cardiovascular diseases** | ESC | 2013 | 238 | 162 | 51 (61.4%) | 20 (24.1%) | 6 (7.2%) | 6 (7.2%) | 37 (44.6%) | 26 (31.3%) | 20 (24.1%) | 83 |
| **2013 ESH/ESC Guidelines for the Management of Arterial Hypertension** | ESC | 2013 | 1362 | 396 | 54 (49.1%) | 30 (27.3%) | 14 (12.7%) | 12 (10.9%) | 35 (31.8%) | 43 (39.1%) | 32 (29.1%) | 110 |
| **2014 AHA/ACC/HRS Guideline for the Management of Patients With Atrial Fibrillation** | ACC/AHA | 2014 | 1473 | 392 | 42 (47.2%) | 22 (24.7%) | 15 (16.9%) | 10 (11.2%) | 6 (6.7%) | 37 (41.6%) | 46 (51.7%) | 89 |
| **2014 AHA/ACC Guideline for the Management of Patients with Non-ST-Elevation Acute Coronary Syndromes** | ACC/AHA | 2014 | 629 | 348 | 54 (63.5%) | 13 (15.3%) | 9 (10.6%) | 9 (10.6%) | 17 (20%) | 41 (48.2%) | 27 (31.8%) | 85 |
| **2014 ACC/AHA Guideline on Perioperative Cardiovascular Evaluation and Management of Patients Undergoing Noncardiac Surgery** | ACC/AHA | 2014 | 314 | 161 | 16 (22.5%) | 18 (25.3%) | 20 (28.2%) | 17 (23.9%) | 3 (4.2%) | 39 (54.9%) | 29 (40.8%) | 71 |
| **2014 ESC/EACTS Guidelines on myocardial revascularization** | ESC | 2014 | 537 | 391 | 150 (46.7%) | 99 (30.8%) | 37 (11.5%) | 35 (10.9%) | 90 (28%) | 108 (33.6%) | 123 (38.3%) | 321 |
| **2014 ESC Guidelines on diagnosis and management of hypertrophic cardiomyopathy** | ESC | 2014 | 424 | 302 | 54 (40.9%) | 46 (34.8%) | 29 (22%) | 3 (2.3%) | 0 (0%) | 36 (27.3%) | 96 (72.7%) | 132 |
| **2014 ESC Guidelines on the diagnosis and treatment of aortic diseases** | ESC | 2014 | 482 | 228 | 66 (55.5%) | 35 (29.4%) | 14 (11.8%) | 4 (3.4%) | 4 (3.4%) | 18 (15.1%) | 97 (81.5%) | 119 |
| **2015 ACC/AHA/HRS Guideline for the Management of Adult Patients With Supraventricular Tachycardia** | ACC/AHA | 2015 | 135 | 69 | 51 (40.8%) | 39 (31.2%) | 32 (25.6%) | 3 (2.4%) | 1 (0.8%) | 68 (54.4%) | 56 (44.8%) | 125 |
| **2015 ESC Guidelines for the management of patients with ventricular arrhythmias and the prevention of sudden cardiac death** | ESC | 2015 | 590 | 334 | 124 (50.4%) | 71 (28.9%) | 37 (15%) | 14 (5.7%) | 15 (6.1%) | 95 (38.6%) | 136 (55.3%) | 246 |
| **2015 ESC Guidelines for the management of acute coronary syndromes in patients presenting without persistent ST-segment elevation** | ESC | 2015 | 892 | 432 | 79 (59%) | 32 (23.9%) | 16 (11.9%) | 7 (5.2%) | 36 (26.9%) | 44 (32.8%) | 54 (40.3%) | 134 |
| **2015 ESC Guidelines for the management of infective endocarditis** | ESC | 2015 | 594 | 331 | 54 (54%) | 26 (26%) | 12 (12%) | 8 (8%) | 1 (1%) | 48 (48%) | 51 (51%) | 100 |
| **2015 ESC Guidelines for the diagnosis and management of pericardial diseases** | ESC | 2015 | 281 | 122 | 55 (49.1%) | 35 (31.3%) | 13 (11.6%) | 9 (8%) | 5 (4.5%) | 23 (20.5%) | 84 (75%) | 112 |
| **2015 ESC/ERS Guidelines for the diagnosis and treatment of pulmonary hypertension** | ESC | 2015 | 759 | 724 | 87 (41.2%) | 46 (21.8%) | 59 (28%) | 19 (9%) | 8 (3.8%) | 41 (19.4%) | 162 (76.8%) | 211 |
| **2016 ACC/AHA Guideline Focused Update on Duration of Dual Antiplatelet Therapy in Patients With Coronary Artery Disease** | ACC/AHA | 2016 | 365 | 276 | 22 (44.9%) | 8 (16.3%) | 14 (28.6%) | 5 (10.2%) | 10 (20.4%) | 28 (57.1%) | 11 (22.4%) | 49 |
| **2016 AHA/ACC Guideline on the Management of Patients With Lower Extremity Peripheral Artery Disease** | ACC/AHA | 2016 | 475 | 291 | 42 (47.7%) | 25 (28.4%) | 11 (12.5%) | 10 (11.4%) | 16 (18.2%) | 40 (45.5%) | 32 (36.4%) | 88 |
| **2016 ESC Guidelines for the management of atrial fibrillation** | ESC | 2016 | 1530 | 860 | 47 (30.5%) | 78 (50.6%) | 16 (10.4%) | 13 (8.4%) | 22 (14.3%) | 80 (51.9%) | 52 (33.8%) | 154 |
| **2016 ESC Guidelines for the diagnosis and treatment of acute and chronic heart failure** | ESC | 2016 | 2950 | 1803 | 79 (42.2%) | 44 (23.5%) | 39 (20.9%) | 25 (13.4%) | 38 (20.3%) | 57 (30.5%) | 92 (49.2%) | 187 |
| **2016 European Guidelines on cardiovascular disease prevention in clinical practice** | ESC | 2016 | 1263 | 732 | 105 (55.6%) | 47 (24.9%) | 22 (11.6%) | 15 (7.9%) | 56 (29.6%) | 84 (44.4%) | 49 (25.9%) | 189 |
| **2016 ACC/AHA/HFSA Focused Update on New Pharmacological Therapy for Heart Failure** | ACC/AHA | 2017 | 830 | 466 | 14 (43.8%) | 7 (21.9%) | 5 (15.6%) | 6 (18.8%) | 7 (21.9%) | 16 (50%) | 9 (28.1%) | 32 |
| **2017 AHA/ACC Focused Update of the 2014 AHA/ACC Guideline for the Management of Patients With Valvular Heart Disease** | ACC/AHA | 2017 | 728 | 121 | 31 (44.3%) | 24 (34.3%) | 12 (17.1%) | 3 (4.3%) | 4 (5.7%) | 44 (62.9%) | 22 (31.4%) | 70 |
| **2017 ACC/AHA/HRS Guideline for the Evaluation and Management of Patients With Syncope** | ACC/AHA | 2017 | 190 | 94 | 26 (25.2%) | 41 (39.8%) | 25 (24.2%) | 11 (10.7%) | 0 (0%) | 55 (53.4%) | 48 (46.6%) | 103 |
| **2017 AHA/ACC/HRS Guideline for Management of Patients With Ventricular Arrhythmias and the Prevention of Sudden Cardiac Death** | ACC/AHA | 2017 | 561 | 308 | 85 (46.4%) | 59 (32.2%) | 21 (11.5%) | 18 (9.8%) | 11 (6.0%) | 134 (73.2%) | 38 (20.8%) | 183 |
| **2017 ACC/AHA/AAPA/ABC/ACPM/AGS/APhA/ASH/ASPC/NMA/PCNA Guideline for the Prevention, Detection, Evaluation, and Management of High Blood Pressure in Adults** | ACC/AHA | 2017 | 1473 | 923 | 68 (58.6%) | 24 (20.7%) | 17 (14.7%) | 7 (6.0%) | 21 (18.1%) | 49 (42.2%) | 46 (39.7%) | 116 |
| **2017 ESC focused update on dual antiplatelet therapy in coronary artery disease** | ESC | 2017 | 467 | 176 | 22 (34.4%) | 20 (31.3%) | 17 (26.6%) | 5 (7.8%) | 15 (23.4%) | 26 (40.6%) | 23 (35.9%) | 64 |
| **2017 ESC Guidelines for the management of acute myocardial infarction in patients presenting with ST-segment elevation** | ESC | 2017 | 1645 | 317 | 92 (57.5%) | 39 (24.4%) | 13 (8.1%) | 16 (10%) | 37 (23.1%) | 44 (27.5%) | 79 (49.4%) | 160 |
| **2017 ESC/EACTS Guidelines for the management of valvular heart disease** | ESC | 2017 | 1148 | 717 | 88 (55.3%) | 48 (30.2%) | 15 (9.4%) | 8 (5%) | 2 (1.3%) | 34 (21.4%) | 123 (77.4%) | 159 |
| **2017 ESC Guidelines on the Diagnosis and Treatment of Peripheral Arterial Diseases** | ESC | 2017 | 609 | 340 | 53 (41.7%) | 40 (31.5%) | 24 (18.9%) | 10 (7.9%) | 20 (15.7%) | 44 (34.6%) | 63 (49.6%) | 127 |
| **2018 ACC/AHA/HRS Guideline on the Evaluation and Management of Patients With Bradycardia and Cardiac Conduction Delay** | ACC/AHA | 2018 | 265 | 110 | 46 (33.1%) | 47 (33.8%) | 27 (19.4%) | 19 (13.7%) | 2 (1.4%) | 62 (44.6%) | 75 (54%) | 139 |
| **2018 AHA/ACC/AACVPR/AAPA/ABC/ACPM/ADA/AGS/APhA/ASPC/NLA/PCNA Guideline on the Management of Blood Cholesterol** | ACC/AHA | 2018 | 890 | 670 | 28 (38.9%) | 26 (36.1%) | 15 (20.8%) | 3 (4.2%) | 11 (15.3%) | 48 (66.7%) | 13 (18.1%) | 72 |
| **2018 AHA/ACC Guideline for the Management of Adults With Congenital Heart Disease** | ACC/AHA | 2018 | 446 | 200 | 90 (50.3%) | 61 (34.1%) | 23 (12.8%) | 5 (2.8%) | 1 (0.6%) | 87 (48.6%) | 91 (50.8%) | 179 |
| **2018 ESC Guidelines for the diagnosis and management of syncope** | ESC | 2018 | 321 | 112 | 46 (40.4%) | 42 (36.8%) | 22 (19.3%) | 4 (3.5%) | 5 (4.4%) | 51 (44.7%) | 58 (50.9%) | 114 |
| **2018 ESC/EACTS Guidelines on myocardial revascularization** | ESC | 2018 | 1410 | 624 | 110 (46.2%) | 74 (31.1%) | 38 (16%) | 16 (6.7%) | 60 (25.2%) | 82 (34.5%) | 96 (40.3%) | 238 |
| **2018 ESC Guidelines for the management of cardiovascular diseases during pregnancy** | ESC | 2018 | 654 | 197 | 80 (54.1%) | 44 (29.7%) | 11 (7.4%) | 13 (8.8%) | 2 (1.4%) | 11 (7.4%) | 135 (91.2%) | 148 |
| **2018 ESC/ESH Guidelines for the management of arterial hypertension** | ESC | 2018 | 2467 | 1220 | 87 (64.4%) | 21 (15.6%) | 14 (10.4%) | 13 (9.6%) | 49 (36.3%) | 39 (28.9%) | 47 (34.8%) | 135 |
| **2019 AHA/ACC/HRS Focused Update of the 2014 AHA/ACC/HRS Guideline for the Management of Patients With Atrial Fibrillation** | ACC/AHA | 2019 | 993 | 496 | 26 (55.3%) | 9 (19.1%) | 10 (21.3%) | 2 (4.3%) | 3 (6.4%) | 31 (66.0%) | 13 (27.7%) | 47 |
| **2019 ACC/AHA Guideline on the Primary Prevention of Cardiovascular Disease** | ACC/AHA | 2019 | 1491 | 1834 | 30 (55.6%) | 13 (24.1%) | 7 (13.0%) | 4 (7.4%) | 12 (22.2%) | 33 (61.1%) | 9 (16.7%) | 54 |
| **2019 ESC Guidelines for the management of patients with supraventricular tachycardia** | ESC | 2019 | 213 | 111 | 51 (41.5%) | 46 (37.4%) | 19 (15.4%) | 7 (5.7%) | 2 (1.6%) | 66 (53.7%) | 55 (44.7%) | 123 |
| **2019 ESC Guidelines for the diagnosis and management of chronic coronary syndromes** | ESC | 2019 | 1437 | 830 | 87 (55.1%) | 28 (17.7%) | 26 (16.5%) | 17 (10.8%) | 45 (28.5%) | 55 (34.8%) | 58 (36.7%) | 158 |
| **2019 ESC/EAS Guidelines for the management of dyslipidaemias** | ESC | 2019 | 1745 | 1091 | 36 (52.2%) | 17 (24.6%) | 11 (15.9%) | 5 (7.2%) | 19 (27.5%) | 13 (18.8%) | 37 (53.6%) | 69 |
| **2019 ESC Guidelines on diabetes, pre-diabetes, and cardiovascular diseases** | ESC | 2019 | 939 | 354 | 78 (56.5%) | 33 (23.9%) | 14 (10.1%) | 13 (9.4%) | 60 (43.5%) | 38 (27.5%) | 40 (29%) | 138 |
| **2019 ESC Guidelines for the diagnosis and management of acute pulmonary embolism** | ESC | 2019 | 941 | 599 | 30 (39.5%) | 31 (40.8%) | 6 (7.9%) | 9 (11.8%) | 16 (21.1%) | 29 (38.2%) | 31 (40.8%) | 76 |
| **2020 AHA/ACC Guideline for the Diagnosis and Treatment of Patients With Hypertrophic Cardiomyopathy** | ACC/AHA | 2020 | 456 | 187 | 75 (55.1%) | 32 (23.5%) | 20 (14.7%) | 9 (6.6%) | 0 (0%) | 78 (57.4%) | 58 (42.6%) | 136 |
| **2020 ACC/AHA Guideline for the Management of Patients With Valvular Heart Disease** | ACC/AHA | 2020 | 1359 | 587 | 122 (44.9%) | 92 (33.8%) | 41 (15.1%) | 17 (6.3%) | 14 (5.1%) | 167 (61.4%) | 91 (33.5%) | 272 |
| **2020 ESC Guidelines for the diagnosis and management of atrial fibrillation** | ESC | 2020 | 2367 | 663 | 63 (48.5%) | 45 (34.6%) | 13 (10.0%) | 9 (6.9%) | 24 (18.5%) | 68 (52.3%) | 38 (29.2%) | 130 |
| **2020 ESC Guidelines for the management of acute coronary syndromes in patients presenting without persistent ST-segment elevation** | ESC | 2020 | 1354 | 575 | 79 (56%) | 35 (24.8%) | 19 (13.5%) | 8 (5.7%) | 39 (27.7%) | 58 (41.1%) | 44 (31.2%) | 141 |
| **2020 ESC Guidelines on sports cardiology and exercise in patients with cardiovascular disease** | ESC | 2020 | 373 | 151 | 52 (32.7%) | 44 (27.7%) | 32 (20.1%) | 31 (19.5%) | 12 (7.5%) | 21 (13.2%) | 126 (79.2%) | 159 |
| **2020 ESC Guidelines for the management of adult congenital heart disease** | ESC | 2020 | 419 | 45 | 59 (43.7%) | 52 (38.5%) | 18 (13.3%) | 6 (4.4%) | 1 (0.7%) | 3 (2.2%) | 131 (97%) | 135 |
| **2021 ACC/AHA/SCAI Guideline for Coronary Artery Revascularization** | ACC/AHA | 2021 | 758 | 368 | 52 (38.5%) | 40 (29.6%) | 24 (17.8%) | 19 (14.1%) | 17 (12.6%) | 85 (63%) | 33 (24.4%) | 135 |
| **2021 AHA/ACC/ASE/CHEST/SAEM/SCCT/SCMR Guideline for the Evaluation and Diagnosis of Chest Pain** | ACC/AHA | 2021 | 500 | 320 | 62 (60.2%) | 35 (34%) | 4 (3.9%) | 2 (1.9%) | 9 (8.7%) | 52 (50.5%) | 42 (40.8%) | 103 |
| **2021 ESC Guidelines on cardiac pacing and cardiac resynchronization therapy** | ESC | 2021 | 582 | 356 | 34 (28.8%) | 46 (39%) | 26 (22%) | 12 (10.2%) | 13 (11%) | 36 (30.5%) | 69 (58.5%) | 118 |
| **2021 ESC Guidelines for the diagnosis and treatment of acute and chronic heart failure** | ESC | 2021 | 3490 | 988 | 52 (43%) | 37 (30.6%) | 26 (21.5%) | 6 (5%) | 25 (20.7%) | 38 (31.4%) | 58 (47.9%) | 121 |
| **2021 ESC Guidelines on cardiovascular disease prevention in clinical practice** | ESC | 2021 | 1528 | 146 | 102 (58%) | 38 (21.6%) | 28 (15.9%) | 8 (4.5%) | 65 (36.9%) | 68 (38.6%) | 43 (24.4%) | 176 |
| **2021 ESC/EACTS Guidelines for the management of valvular heart disease** | ESC | 2021 | 1441 | 907 | 55 (46.2%) | 46 (38.7%) | 14 (11.8%) | 4 (3.4%) | 4 (3.4%) | 45 (37.8%) | 70 (58.8%) | 119 |
| **2022 AHA/ACC/HFSA Guideline for the Management of Heart Failure** | ACC/AHA | 2022 | 2069 | 1674 | 81 (45.8%) | 50 (28.2%) | 21 (11.9%) | 25 (14.1%) | 26 (14.7%) | 105 (59.3%) | 46 (26.0%) | 177 |
| **2022 ACC/AHA Guideline for the Diagnosis and Management of Aortic Disease** | ACC/AHA | 2022 | 434 | 263 | 147 (51.6%) | 86 (30.2%) | 50 (17.5%) | 2 (0.7%) | 5 (1.8%) | 105 (36.8%) | 175 (61.4%) | 285 |
| **2022 ESC Guidelines for the management of patients with ventricular arrhythmias and the prevention of sudden cardiac death** | ESC | 2022 | 673 | 409 | 140 (42.8%) | 117 (35.6%) | 50 (15.3%) | 20 (6.1%) | 12 (3.7%) | 96 (29.4%) | 219 (67.0%) | 327 |
| **2022 ESC Guidelines on cardiovascular assessment and management of patients undergoing non-cardiac surgery** | ESC | 2022 | 272 | 151 | 75 (51.4%) | 42 (28.8%) | 15 (10.3%) | 14 (9.6%) | 17 (11.6%) | 52 (35.6%) | 77 (52.7%) | 146 |
| **2022 ESC Guidelines on cardio-oncology** | ESC | 2022 | 689 | 207 | 156 (57.4%) | 75 (27.6%) | 36 (13.2%) | 5 (1.8%) | 7 (2.6%) | 57 (21.0%) | 208 (76.5%) | 272 |
| **2022 ESC/ERS Guidelines for the diagnosis and treatment of pulmonary hypertension** | ESC | 2022 | 1256 | 797 | 98 (54.4%) | 37 (20.6%) | 26 (14.4%) | 19 (10.6%) | 7 (3.9%) | 44 (24.4%) | 129 (71.7%) | 180 |
| **2023 ACC/AHA/ACCP/HRS Guideline for the Diagnosis and Management of Atrial Fibrillation** | ACC/AHA | 2023 | 418 | 230 | 75 (38.7%) | 65 (33.5%) | 36 (18.6%) | 18 (9.3%) | 33 (17%) | 128 (66%) | 33 (17%) | 194 |
| **2023 AHA/ACC/ACCP/ASPC/NLA/PCNA Guideline for the Management of Patients With Chronic Coronary Disease** | ACC/AHA | 2023 | 348 | 158 | 57 (42.2%) | 35 (25.9%) | 22 (16.3%) | 21 (15.6%) | 30 (22.2%) | 79 (58.5%) | 26 (19.3%) | 135 |
| **2023 ESC Guidelines for the management of acute coronary syndromes** | ESC | 2023 | 1032 | 622 | 107 (55.4%) | 48 (24.9%) | 21 (10.9%) | 17 (8.8%) | 56 (29%) | 64 (33.2%) | 73 (37.8%) | 193 |
| **2023 ESC Guidelines for the management of endocarditis** | ESC | 2023 | 374 | 207 | 72 (60%) | 25 (20.8%) | 17 (14.2%) | 6 (5%) | 4 (3.3%) | 49 (40.8%) | 67 (55.8%) | 120 |
| **2023 ESC Guidelines for the management of cardiomyopathies** | ESC | 2023 | 506 | 294 | 71 (44.9%) | 55 (34.8%) | 27 (17.1%) | 5 (3.2%) | 5 (3.2%) | 51 (32.3%) | 102 (64.6%) | 158 |
| **2023 ESC Guidelines for the management of cardiovascular disease in patients with diabetes** | ESC | 2023 | 367 | 189 | 95 (66%) | 32 (22.2%) | 12 (8.3%) | 5 (3.5%) | 56 (38.9%) | 51 (35.4%) | 37 (25.7%) | 144 |
| **2024 AHA/ACC/AMSSM/HRS/PACES/SCMR Guideline for the Management of Hypertrophic Cardiomyopathy** | ACC/AHA | 2024 | - | - | 77 (54.6%) | 32 (22.7%) | 21 (14.9%) | 11 (7.8%) | 0 (0%) | 87 (61.7%) | 54 (38.3%) | 141 |
| **2024 ACC/AHA/AACVPR/APMA/ABC/SCAI/SVM/SVN/SVS/SIR/VESS Guideline for the Management of Lower Extremity Peripheral Artery Disease** | ACC/AHA | 2024 | - | - | 65 (52%) | 29 (23.2%) | 19 (15.2%) | 12 (9.6%) | 20 (16%) | 61 (48.8%) | 44 (35.2%) | 125 |
| **2024 ESC Guidelines for the management of atrial fibrillation** | ESC | 2024 | - | - | 65 (49.6%) | 40 (30.5%) | 12 (9.2%) | 14 (10.7%) | 24 (18.3%) | 66 (50.4%) | 41 (31.3%) | 131 |
| **2024 ESC Guidelines for the management of chronic coronary syndromes** | ESC | 2024 | - | - | 124 (63.6%) | 37 (19%) | 24 (12.3%) | 10 (5.1%) | 54 (27.7%) | 92 (47.2%) | 49 (25.1%) | 195 |
| **2024 ESC Guidelines for the management of elevated blood pressure and hypertension** | ESC | 2024 | - | - | 72 (53.7%) | 37 (27.6%) | 18 (13.4%) | 7 (5.2%) | 34 (25.4%) | 62 (46.3%) | 38 (28.4%) | 134 |
| **2024 ESC Guidelines for the management of peripheral arterial and aortic diseases** | ESC | 2024 | - | - | 190 (51.9%) | 110 (30.1%) | 47 (12.8%) | 19 (5.2%) | 45 (12.3%) | 136 (37.2%) | 185 (50.5%) | 366 |

**Supplementary Table 4** – AGREE II 3^rd^ domain scores of the included guidelines.

| **Guideline** | **Soc.** | **Year** | **AGREE II instrument, domain 3** | | | | | | | | |
| --- | --- | --- | --- | --- | --- | --- | --- | --- | --- | --- | --- |
|  |  |  | **point 7** | **point 8** | **point 9** | **point 10** | **point 11** | **point 12** | **point 13** | **point 14** | **Overall** |
| **2013 ACCF/AHA Guideline for the Management of ST-Elevation Myocardial Infarction** | ACC/AHA | 2013 | 5 | 3 | 2 | 4 | 6 | 6 | 2 | 2 | 30 |
| **2013 ACCF/AHA Guideline for the Management of Heart Failure** | ACC/AHA | 2013 | 6 | 3 | 2 | 4 | 7 | 6 | 2 | 2 | 32 |
| **2013 ACC/AHA Guideline on the Treatment of Blood Cholesterol to Reduce Atherosclerotic Cardiovascular Risk in Adults** | ACC/AHA | 2013 | 6 | 7 | 7 | 4 | 7 | 7 | 3 | 1 | 42 |
| **2013 ESC guidelines on the management of stable coronary artery disease** | ESC | 2013 | 1 | 1 | 2 | 4 | 6 | 6 | 2 | 1 | 23 |
| **2013 ESC Guidelines on diabetes, pre-diabetes, and cardiovascular diseases** | ESC | 2013 | 1 | 1 | 2 | 4 | 6 | 6 | 2 | 1 | 23 |
| **2013 ESH/ESC Guidelines for the Management of Arterial Hypertension** | ESC | 2013 | 1 | 1 | 2 | 4 | 6 | 6 | 2 | 1 | 23 |
| **2014 AHA/ACC/HRS Guideline for the Management of Patients With Atrial Fibrillation** | ACC/AHA | 2014 | 7 | 2 | 2 | 5 | 7 | 7 | 3 | 1 | 34 |
| **2014 AHA/ACC Guideline for the Management of Patients with Non-ST-Elevation Acute Coronary Syndromes** | ACC/AHA | 2014 | 6 | 2 | 5 | 4 | 6 | 6 | 2 | 4 | 35 |
| **2014 ACC/AHA Guideline on Perioperative Cardiovascular Evaluation and Management of Patients Undergoing Noncardiac Surgery** | ACC/AHA | 2014 | 6 | 3 | 5 | 4 | 6 | 6 | 2 | 4 | 36 |
| **2014 ESC/EACTS Guidelines on myocardial revascularization** | ESC | 2014 | 1 | 1 | 2 | 4 | 6 | 6 | 2 | 1 | 23 |
| **2014 ESC Guidelines on diagnosis and management of hypertrophic cardiomyopathy** | ESC | 2014 | 1 | 1 | 2 | 4 | 6 | 6 | 3 | 1 | 24 |
| **2014 ESC Guidelines on the diagnosis and treatment of aortic diseases** | ESC | 2014 | 1 | 1 | 2 | 4 | 6 | 6 | 2 | 1 | 23 |
| **2015 ACC/AHA/HRS Guideline for the Management of Adult Patients With Supraventricular Tachycardia** | ACC/AHA | 2015 | 6 | 5 | 6 | 4 | 6 | 6 | 2 | 4 | 39 |
| **2015 ESC Guidelines for the management of patients with ventricular arrhythmias and the prevention of sudden cardiac death** | ESC | 2015 | 1 | 1 | 2 | 4 | 6 | 6 | 2 | 1 | 23 |
| **2015 ESC Guidelines for the management of acute coronary syndromes in patients presenting without persistent ST-segment elevation** | ESC | 2015 | 1 | 1 | 2 | 4 | 6 | 6 | 2 | 1 | 23 |
| **2015 ESC Guidelines for the management of infective endocarditis** | ESC | 2015 | 1 | 1 | 2 | 4 | 6 | 6 | 2 | 1 | 23 |
| **2015 ESC Guidelines for the diagnosis and management of pericardial diseases** | ESC | 2015 | 1 | 1 | 2 | 4 | 6 | 6 | 2 | 1 | 23 |
| **2015 ESC/ERS Guidelines for the diagnosis and treatment of pulmonary hypertension** | ESC | 2015 | 1 | 1 | 2 | 4 | 6 | 6 | 2 | 1 | 23 |
| **2016 ACC/AHA Guideline Focused Update on Duration of Dual Antiplatelet Therapy in Patients With Coronary Artery Disease** | ACC/AHA | 2016 | 3 | 3 | 7 | 4 | 7 | 6 | 2 | 2 | 34 |
| **2016 AHA/ACC Guideline on the Management of Patients With Lower Extremity Peripheral Artery Disease** | ACC/AHA | 2016 | 6 | 3 | 5 | 4 | 6 | 7 | 2 | 4 | 37 |
| **2016 ESC Guidelines for the management of atrial fibrillation** | ESC | 2016 | 1 | 1 | 2 | 4 | 6 | 6 | 2 | 1 | 23 |
| **2016 ESC Guidelines for the diagnosis and treatment of acute and chronic heart failure** | ESC | 2016 | 1 | 1 | 2 | 4 | 6 | 6 | 2 | 1 | 23 |
| **2016 European Guidelines on cardiovascular disease prevention in clinical practice** | ESC | 2016 | 1 | 1 | 2 | 4 | 6 | 6 | 2 | 1 | 23 |
| **2016 ACC/AHA/HFSA Focused Update on New Pharmacological Therapy for Heart Failure** | ACC/AHA | 2017 | 5 | 3 | 5 | 4 | 7 | 7 | 2 | 4 | 37 |
| **2017 AHA/ACC Focused Update of the 2014 AHA/ACC Guideline for the Management of Patients With Valvular Heart Disease** | ACC/AHA | 2017 | 3 | 3 | 5 | 4 | 7 | 7 | 2 | 4 | 35 |
| **2017 ACC/AHA/HRS Guideline for the Evaluation and Management of Patients With Syncope** | ACC/AHA | 2017 | 6 | 5 | 6 | 4 | 6 | 7 | 2 | 4 | 40 |
| **2017 AHA/ACC/HRS Guideline for Management of Patients With Ventricular Arrhythmias and the Prevention of Sudden Cardiac Death** | ACC/AHA | 2017 | 6 | 5 | 5 | 4 | 6 | 7 | 2 | 4 | 39 |
| **2017 ACC/AHA/AAPA/ABC/ACPM/AGS/APhA/ASH/ASPC/NMA/PCNA Guideline for the Prevention, Detection, Evaluation, and Management of High Blood Pressure in Adults** | ACC/AHA | 2017 | 7 | 7 | 6 | 4 | 6 | 7 | 2 | 4 | 43 |
| **2017 ESC focused update on dual antiplatelet therapy in coronary artery disease** | ESC | 2017 | 1 | 1 | 2 | 4 | 7 | 6 | 2 | 1 | 24 |
| **2017 ESC Guidelines for the management of acute myocardial infarction in patients presenting with ST-segment elevation** | ESC | 2017 | 1 | 1 | 2 | 4 | 6 | 6 | 2 | 1 | 23 |
| **2017 ESC/EACTS Guidelines for the management of valvular heart disease** | ESC | 2017 | 1 | 1 | 2 | 4 | 6 | 6 | 2 | 1 | 23 |
| **2017 ESC Guidelines on the Diagnosis and Treatment of Peripheral Arterial Diseases** | ESC | 2017 | 1 | 1 | 2 | 4 | 6 | 6 | 2 | 1 | 23 |
| **2018 ACC/AHA/HRS Guideline on the Evaluation and Management of Patients With Bradycardia and Cardiac Conduction Delay** | ACC/AHA | 2018 | 7 | 7 | 6 | 4 | 6 | 7 | 2 | 4 | 43 |
| **2018 AHA/ACC/AACVPR/AAPA/ABC/ACPM/ADA/AGS/APhA/ASPC/NLA/PCNA Guideline on the Management of Blood Cholesterol** | ACC/AHA | 2018 | 7 | 7 | 6 | 4 | 7 | 7 | 2 | 1 | 41 |
| **2018 AHA/ACC Guideline for the Management of Adults With Congenital Heart Disease** | ACC/AHA | 2018 | 7 | 7 | 6 | 4 | 6 | 7 | 2 | 4 | 43 |
| **2018 ESC Guidelines for the diagnosis and management of syncope** | ESC | 2018 | 1 | 1 | 2 | 4 | 7 | 6 | 2 | 1 | 24 |
| **2018 ESC/EACTS Guidelines on myocardial revascularization** | ESC | 2018 | 1 | 1 | 2 | 4 | 6 | 6 | 2 | 1 | 23 |
| **2018 ESC Guidelines for the management of cardiovascular diseases during pregnancy** | ESC | 2018 | 2 | 1 | 2 | 4 | 6 | 6 | 2 | 1 | 24 |
| **2018 ESC/ESH Guidelines for the management of arterial hypertension** | ESC | 2018 | 1 | 1 | 2 | 4 | 6 | 6 | 2 | 1 | 23 |
| **2019 AHA/ACC/HRS Focused Update of the 2014 AHA/ACC/HRS Guideline for the Management of Patients With Atrial Fibrillation** | ACC/AHA | 2019 | 6 | 2 | 5 | 4 | 6 | 7 | 2 | 4 | 36 |
| **2019 ACC/AHA Guideline on the Primary Prevention of Cardiovascular Disease** | ACC/AHA | 2019 | 6 | 2 | 5 | 4 | 6 | 7 | 2 | 1 | 33 |
| **2019 ESC Guidelines for the management of patients with supraventricular tachycardia** | ESC | 2019 | 1 | 1 | 2 | 4 | 6 | 6 | 2 | 1 | 23 |
| **2019 ESC Guidelines for the diagnosis and management of chronic coronary syndromes** | ESC | 2019 | 1 | 1 | 2 | 4 | 6 | 6 | 2 | 1 | 23 |
| **2019 ESC/EAS Guidelines for the management of dyslipidaemias** | ESC | 2019 | 1 | 1 | 2 | 4 | 6 | 6 | 2 | 1 | 23 |
| **2019 ESC Guidelines on diabetes, pre-diabetes, and cardiovascular diseases** | ESC | 2019 | 1 | 1 | 2 | 4 | 6 | 6 | 2 | 1 | 23 |
| **2019 ESC Guidelines for the diagnosis and management of acute pulmonary embolism** | ESC | 2019 | 1 | 1 | 2 | 4 | 7 | 6 | 2 | 1 | 24 |
| **2020 AHA/ACC Guideline for the Diagnosis and Treatment of Patients With Hypertrophic Cardiomyopathy** | ACC/AHA | 2020 | 6 | 2 | 5 | 4 | 6 | 7 | 2 | 2 | 34 |
| **2020 ACC/AHA Guideline for the Management of Patients With Valvular Heart Disease** | ACC/AHA | 2020 | 6 | 2 | 5 | 4 | 6 | 7 | 2 | 2 | 34 |
| **2020 ESC Guidelines for the diagnosis and management of atrial fibrillation** | ESC | 2020 | 1 | 1 | 2 | 4 | 7 | 6 | 2 | 1 | 24 |
| **2020 ESC Guidelines for the management of acute coronary syndromes in patients presenting without persistent ST-segment elevation** | ESC | 2020 | 1 | 1 | 2 | 4 | 7 | 6 | 2 | 1 | 24 |
| **2020 ESC Guidelines on sports cardiology and exercise in patients with cardiovascular disease** | ESC | 2020 | 1 | 1 | 2 | 4 | 6 | 6 | 2 | 1 | 23 |
| **2020 ESC Guidelines for the management of adult congenital heart disease** | ESC | 2020 | 1 | 1 | 2 | 4 | 6 | 6 | 2 | 1 | 23 |
| **2021 ACC/AHA/SCAI Guideline for Coronary Artery Revascularization** | ACC/AHA | 2021 | 6 | 2 | 5 | 4 | 6 | 7 | 2 | 2 | 34 |
| **2021 AHA/ACC/ASE/CHEST/SAEM/SCCT/SCMR Guideline for the Evaluation and Diagnosis of Chest Pain** | ACC/AHA | 2021 | 6 | 2 | 5 | 4 | 6 | 7 | 2 | 2 | 34 |
| **2021 ESC Guidelines on cardiac pacing and cardiac resynchronization therapy** | ESC | 2021 | 1 | 1 | 2 | 4 | 7 | 6 | 2 | 1 | 24 |
| **2021 ESC Guidelines for the diagnosis and treatment of acute and chronic heart failure** | ESC | 2021 | 1 | 1 | 2 | 4 | 7 | 6 | 2 | 1 | 24 |
| **2021 ESC Guidelines on cardiovascular disease prevention in clinical practice** | ESC | 2021 | 1 | 1 | 2 | 4 | 6 | 6 | 2 | 1 | 23 |
| **2021 ESC/EACTS Guidelines for the management of valvular heart disease** | ESC | 2021 | 1 | 1 | 2 | 4 | 6 | 6 | 2 | 1 | 23 |
| **2022 AHA/ACC/HFSA Guideline for the Management of Heart Failure** | ACC/AHA | 2022 | 6 | 2 | 5 | 4 | 6 | 7 | 2 | 2 | 34 |
| **2022 ACC/AHA Guideline for the Diagnosis and Management of Aortic Disease** | ACC/AHA | 2022 | 6 | 2 | 5 | 4 | 6 | 7 | 2 | 2 | 34 |
| **2022 ESC Guidelines for the management of patients with ventricular arrhythmias and the prevention of sudden cardiac death** | ESC | 2022 | 3 | 1 | 2 | 4 | 7 | 6 | 2 | 1 | 26 |
| **2022 ESC Guidelines on cardiovascular assessment and management of patients undergoing non-cardiac surgery** | ESC | 2022 | 1 | 1 | 2 | 4 | 6 | 6 | 2 | 1 | 23 |
| **2022 ESC Guidelines on cardio-oncology** | ESC | 2022 | 1 | 1 | 2 | 4 | 6 | 6 | 2 | 1 | 23 |
| **2022 ESC/ERS Guidelines for the diagnosis and treatment of pulmonary hypertension** | ESC | 2022 | 7 | 7 | 7 | 4 | 6 | 6 | 2 | 1 | 40 |
| **2023 ACC/AHA/ACCP/HRS Guideline for the Diagnosis and Management of Atrial Fibrillation** | ACC/AHA | 2023 | 6 | 2 | 5 | 4 | 7 | 7 | 2 | 2 | 35 |
| **2023 AHA/ACC/ACCP/ASPC/NLA/PCNA Guideline for the Management of Patients With Chronic Coronary Disease** | ACC/AHA | 2023 | 6 | 2 | 5 | 4 | 7 | 7 | 2 | 2 | 35 |
| **2023 ESC Guidelines for the management of acute coronary syndromes** | ESC | 2023 | 1 | 1 | 2 | 4 | 7 | 6 | 2 | 1 | 24 |
| **2023 ESC Guidelines for the management of endocarditis** | ESC | 2023 | 1 | 1 | 2 | 4 | 7 | 6 | 2 | 1 | 24 |
| **2023 ESC Guidelines for the management of cardiomyopathies** | ESC | 2023 | 1 | 1 | 2 | 4 | 7 | 6 | 2 | 1 | 24 |
| **2023 ESC Guidelines for the management of cardiovascular disease in patients with diabetes** | ESC | 2023 | 1 | 1 | 2 | 4 | 7 | 6 | 2 | 1 | 24 |
| **2024 AHA/ACC/AMSSM/HRS/PACES/SCMR Guideline for the Management of Hypertrophic Cardiomyopathy** | ACC/AHA | 2024 | 6 | 2 | 5 | 4 | 6 | 7 | 2 | 2 | 34 |
| **2024 ACC/AHA/AACVPR/APMA/ABC/SCAI/SVM/SVN/SVS/SIR/VESS Guideline for the Management of Lower Extremity Peripheral Artery Disease** | ACC/AHA | 2024 | 6 | 2 | 5 | 4 | 7 | 7 | 2 | 2 | 35 |
| **2024 ESC Guidelines for the management of atrial fibrillation** | ESC | 2024 | 1 | 1 | 5 | 4 | 7 | 6 | 2 | 1 | 27 |
| **2024 ESC Guidelines for the management of chronic coronary syndromes** | ESC | 2024 | 1 | 1 | 5 | 4 | 7 | 6 | 2 | 1 | 27 |
| **2024 ESC Guidelines for the management of elevated blood pressure and hypertension** | ESC | 2024 | 1 | 1 | 5 | 4 | 7 | 6 | 2 | 1 | 27 |
| **2024 ESC Guidelines for the management of peripheral arterial and aortic diseases** | ESC | 2024 | 1 | 1 | 5 | 4 | 7 | 6 | 2 | 1 | 27 |

**References for Supplementary Tables 3 and 4**:

1. O'Gara PT, Kushner FG, Ascheim DD, Casey DE Jr, Chung MK, de Lemos JA, Ettinger SM, Fang JC, Fesmire FM, Franklin BA, Granger CB, Krumholz HM, Linderbaum JA, Morrow DA, Newby LK, Ornato JP, Ou N, Radford MJ, Tamis-Holland JE, Tommaso CL, Tracy CM, Woo YJ, Zhao DX. 2013 ACCF/AHA guideline for the management of ST-elevation myocardial infarction: a report of the American College of Cardiology Foundation/American Heart Association Task Force on Practice Guidelines. J Am Coll Cardiol. 2013 Jan 29;61(4):e78-e140.
2. Yancy CW, Jessup M, Bozkurt B, Butler J, Casey DE Jr, Drazner MH, Fonarow GC, Geraci SA, Horwich T, Januzzi JL, Johnson MR, Kasper EK, Levy WC, Masoudi FA, McBride PE, McMurray JJ, Mitchell JE, Peterson PN, Riegel B, Sam F, Stevenson LW, Tang WH, Tsai EJ, Wilkoff BL; American College of Cardiology Foundation; American Heart Association Task Force on Practice Guidelines. 2013 ACCF/AHA guideline for the management of heart failure: a report of the American College of Cardiology Foundation/American Heart Association Task Force on Practice Guidelines. J Am Coll Cardiol. 2013 Oct 15;62(16):e147-239.
3. Stone NJ, Robinson JG, Lichtenstein AH, Bairey Merz CN, Blum CB, Eckel RH, Goldberg AC, Gordon D, Levy D, Lloyd-Jones DM, McBride P, Schwartz JS, Shero ST, Smith SC Jr, Watson K, Wilson PW; American College of Cardiology/American Heart Association Task Force on Practice Guidelines. 2013 ACC/AHA guideline on the treatment of blood cholesterol to reduce atherosclerotic cardiovascular risk in adults: a report of the American College of Cardiology/American Heart Association Task Force on Practice Guidelines. J Am Coll Cardiol. 2014 Jul 1;63(25 Pt B):2889-934.
4. Montalescot G, Sechtem U, Achenbach S, Andreotti F, Arden C, Budaj A, Bugiardini R, Crea F, Cuisset T, Di Mario C, Ferreira JR, Gersh BJ, Gitt AK, Hulot JS, Marx N, Opie LH, Pfisterer M, Prescott E, Ruschitzka F, Sabaté M, Senior R, Taggart DP, van der Wall EE, Vrints CJ. 2013 ESC guidelines on the management of stable coronary artery disease: the Task Force on the management of stable coronary artery disease of the European Society of Cardiology. Eur Heart J. 2013 Oct;34(38):2949-3003
5. Rydén L, Grant PJ, Anker SD, Berne C, Cosentino F, Danchin N, Deaton C, Escaned J, Hammes HP, Huikuri H, Marre M, Marx N, Mellbin L, Ostergren J, Patrono C, Seferovic P, Uva MS, Taskinen MR, Tendera M, Tuomilehto J, Valensi P, Zamorano JL. ESC Guidelines on diabetes, pre-diabetes, and cardiovascular diseases developed in collaboration with the EASD: the Task Force on diabetes, pre-diabetes, and cardiovascular diseases of the European Society of Cardiology (ESC) and developed in collaboration with the European Association for the Study of Diabetes (EASD). Eur Heart J. 2013 Oct;34(39):3035-87.
6. Mancia G, Fagard R, Narkiewicz K, Redon J, Zanchetti A, Böhm M, et al. 2013 ESH/ESC guidelines for the management of arterial hypertension: the Task Force for the Management of Arterial Hypertension of the European Society of Hypertension (ESH) and of the European Society of Cardiology (ESC). Eur Heart J. 2013 Jul;34(28):2159-219.
7. January CT, Wann LS, Alpert JS, Calkins H, Cigarroa JE, Cleveland JC Jr, Conti JB, Ellinor PT, Ezekowitz MD, Field ME, Murray KT, Sacco RL, Stevenson WG, Tchou PJ, Tracy CM, Yancy CW; American College of Cardiology/American Heart Association Task Force on Practice Guidelines. 2014 AHA/ACC/HRS guideline for the management of patients with atrial fibrillation: a report of the American College of Cardiology/American Heart Association Task Force on Practice Guidelines and the Heart Rhythm Society. J Am Coll Cardiol. 2014 Dec 2;64(21):e1-76.
8. Amsterdam EA, Wenger NK, Brindis RG, Casey DE Jr, Ganiats TG, Holmes DR Jr, Jaffe AS, Jneid H, Kelly RF, Kontos MC, Levine GN, Liebson PR, Mukherjee D, Peterson ED, Sabatine MS, Smalling RW, Zieman SJ. 2014 AHA/ACC Guideline for the Management of Patients with Non-ST-Elevation Acute Coronary Syndromes: a report of the American College of Cardiology/American Heart Association Task Force on Practice Guidelines. J Am Coll Cardiol. 2014 Dec 23;64(24):e139-e228.
9. Fleisher LA, Fleischmann KE, Auerbach AD, Barnason SA, Beckman JA, Bozkurt B, Davila-Roman VG, Gerhard-Herman MD, Holly TA, Kane GC, Marine JE, Nelson MT, Spencer CC, Thompson A, Ting HH, Uretsky BF, Wijeysundera DN. 2014 ACC/AHA guideline on perioperative cardiovascular evaluation and management of patients undergoing noncardiac surgery: a report of the American College of Cardiology/American Heart Association Task Force on Practice Guidelines. Circulation. 2014 Dec 9;130(24):e278-333.
10. Windecker S, Kolh P, Alfonso F, Collet JP, Cremer J, Falk V, Filippatos G, Hamm C, Head SJ, Jüni P, Kappetein AP, Kastrati A, Knuuti J, Landmesser U, Laufer G, Neumann FJ, Richter DJ, Schauerte P, Sousa Uva M, Stefanini GG, Taggart DP, Torracca L, Valgimigli M, Wijns W, Witkowski A. 2014 ESC/EACTS Guidelines on myocardial revascularization: The Task Force on Myocardial Revascularization of the European Society of Cardiology (ESC) and the European Association for Cardio-Thoracic Surgery (EACTS)Developed with the special contribution of the European Association of Percutaneous Cardiovascular Interventions (EAPCI). Eur Heart J. 2014 Oct 1;35(37):2541-619
11. Elliott PM, Anastasakis A, Borger MA, Borggrefe M, Cecchi F, Charron P, Hagege AA, Lafont A, Limongelli G, Mahrholdt H, McKenna WJ, Mogensen J, Nihoyannopoulos P, Nistri S, Pieper PG, Pieske B, Rapezzi C, Rutten FH, Tillmanns C, Watkins H. 2014 ESC Guidelines on diagnosis and management of hypertrophic cardiomyopathy: the Task Force for the Diagnosis and Management of Hypertrophic Cardiomyopathy of the European Society of Cardiology (ESC). Eur Heart J. 2014 Oct 14;35(39):2733-79.
12. Erbel R, Aboyans V, Boileau C, Bossone E, Bartolomeo RD, Eggebrecht H, Evangelista A, Falk V, Frank H, Gaemperli O, Grabenwöger M, Haverich A, Iung B, Manolis AJ, Meijboom F, Nienaber CA, Roffi M, Rousseau H, Sechtem U, Sirnes PA, Allmen RS, Vrints CJ; ESC Committee for Practice Guidelines. 2014 ESC Guidelines on the diagnosis and treatment of aortic diseases: Document covering acute and chronic aortic diseases of the thoracic and abdominal aorta of the adult. The Task Force for the Diagnosis and Treatment of Aortic Diseases of the European Society of Cardiology (ESC). Eur Heart J. 2014 Nov 1;35(41):2873-926
13. Page RL, Joglar JA, Caldwell MA, Calkins H, Conti JB, Deal BJ, Estes NA 3rd, Field ME, Goldberger ZD, Hammill SC, Indik JH, Lindsay BD, Olshansky B, Russo AM, Shen WK, Tracy CM, Al-Khatib SM; Evidence Review Committee Chair‡. 2015 ACC/AHA/HRS Guideline for the Management of Adult Patients With Supraventricular Tachycardia: Executive Summary: A Report of the American College of Cardiology/American Heart Association Task Force on Clinical Practice Guidelines and the Heart Rhythm Society. Circulation. 2016 Apr 5;133(14):e471-505.
14. Priori SG, Blomström-Lundqvist C, Mazzanti A, Blom N, Borggrefe M, Camm J, Elliott PM, Fitzsimons D, Hatala R, Hindricks G, Kirchhof P, Kjeldsen K, Kuck KH, Hernandez-Madrid A, Nikolaou N, Norekvål TM, Spaulding C, Van Veldhuisen DJ; ESC Scientific Document Group. 2015 ESC Guidelines for the management of patients with ventricular arrhythmias and the prevention of sudden cardiac death: The Task Force for the Management of Patients with Ventricular Arrhythmias and the Prevention of Sudden Cardiac Death of the European Society of Cardiology (ESC). Endorsed by: Association for European Paediatric and Congenital Cardiology (AEPC). Eur Heart J. 2015 Nov 1;36(41):2793-2867.
15. Roffi M, Patrono C, Collet JP, Mueller C, Valgimigli M, Andreotti F, Bax JJ, Borger MA, Brotons C, Chew DP, Gencer B, Hasenfuss G, Kjeldsen K, Lancellotti P, Landmesser U, Mehilli J, Mukherjee D, Storey RF, Windecker S; ESC Scientific Document Group. 2015 ESC Guidelines for the management of acute coronary syndromes in patients presenting without persistent ST-segment elevation: Task Force for the Management of Acute Coronary Syndromes in Patients Presenting without Persistent ST-Segment Elevation of the European Society of Cardiology (ESC). Eur Heart J. 2016 Jan 14;37(3):267-315.
16. Habib G, Lancellotti P, Antunes MJ, Bongiorni MG, Casalta JP, Del Zotti F, Dulgheru R, El Khoury G, Erba PA, Iung B, Miro JM, Mulder BJ, Plonska-Gosciniak E, Price S, Roos-Hesselink J, Snygg-Martin U, Thuny F, Tornos Mas P, Vilacosta I, Zamorano JL; ESC Scientific Document Group. 2015 ESC Guidelines for the management of infective endocarditis: The Task Force for the Management of Infective Endocarditis of the European Society of Cardiology (ESC). Endorsed by: European Association for Cardio-Thoracic Surgery (EACTS), the European Association of Nuclear Medicine (EANM). Eur Heart J. 2015 Nov 21;36(44):3075-3128.
17. Adler Y, Charron P, Imazio M, Badano L, Barón-Esquivias G, Bogaert J, Brucato A, Gueret P, Klingel K, Lionis C, Maisch B, Mayosi B, Pavie A, Ristic AD, Sabaté Tenas M, Seferovic P, Swedberg K, Tomkowski W; ESC Scientific Document Group. 2015 ESC Guidelines for the diagnosis and management of pericardial diseases: The Task Force for the Diagnosis and Management of Pericardial Diseases of the European Society of Cardiology (ESC)Endorsed by: The European Association for Cardio-Thoracic Surgery (EACTS). Eur Heart J. 2015 Nov 7;36(42):2921-2964.
18. Galiè N, Humbert M, Vachiery JL, Gibbs S, Lang I, Torbicki A, Simonneau G, Peacock A, Vonk Noordegraaf A, Beghetti M, Ghofrani A, Gomez Sanchez MA, Hansmann G, Klepetko W, Lancellotti P, Matucci M, McDonagh T, Pierard LA, Trindade PT, Zompatori M, Hoeper M; ESC Scientific Document Group. 2015 ESC/ERS Guidelines for the diagnosis and treatment of pulmonary hypertension: The Joint Task Force for the Diagnosis and Treatment of Pulmonary Hypertension of the European Society of Cardiology (ESC) and the European Respiratory Society (ERS): Endorsed by: Association for European Paediatric and Congenital Cardiology (AEPC), International Society for Heart and Lung Transplantation (ISHLT). Eur Heart J. 2016 Jan 1;37(1):67-119.
19. Levine GN, Bates ER, Bittl JA, Brindis RG, Fihn SD, Fleisher LA, Granger CB, Lange RA, Mack MJ, Mauri L, Mehran R, Mukherjee D, Newby LK, O'Gara PT, Sabatine MS, Smith PK, Smith SC Jr. 2016 ACC/AHA Guideline Focused Update on Duration of Dual Antiplatelet Therapy in Patients With Coronary Artery Disease: A Report of the American College of Cardiology/American Heart Association Task Force on Clinical Practice Guidelines. J Am Coll Cardiol. 2016 Sep 6;68(10):1082-115.
20. Gerhard-Herman MD, Gornik HL, Barrett C, Barshes NR, Corriere MA, Drachman DE, Fleisher LA, Fowkes FGR, Hamburg NM, Kinlay S, Lookstein R, Misra S, Mureebe L, Olin JW, Patel RAG, Regensteiner JG, Schanzer A, Shishehbor MH, Stewart KJ, Treat-Jacobson D, Walsh ME. 2016 AHA/ACC Guideline on the Management of Patients With Lower Extremity Peripheral Artery Disease: A Report of the American College of Cardiology/American Heart Association Task Force on Clinical Practice Guidelines. J Am Coll Cardiol. 2017 Mar 21;69(11):e71-e126.
21. Kirchhof P, Benussi S, Kotecha D, Ahlsson A, Atar D, Casadei B, Castella M, Diener HC, Heidbuchel H, Hendriks J, Hindricks G, Manolis AS, Oldgren J, Popescu BA, Schotten U, Van Putte B, Vardas P; ESC Scientific Document Group. 2016 ESC Guidelines for the management of atrial fibrillation developed in collaboration with EACTS. Eur Heart J. 2016 Oct 7;37(38):2893-2962.
22. Ponikowski P, Voors AA, Anker SD, Bueno H, Cleland JGF, Coats AJS, Falk V, González-Juanatey JR, Harjola VP, Jankowska EA, Jessup M, Linde C, Nihoyannopoulos P, Parissis JT, Pieske B, Riley JP, Rosano GMC, Ruilope LM, Ruschitzka F, Rutten FH, van der Meer P; ESC Scientific Document Group. 2016 ESC Guidelines for the diagnosis and treatment of acute and chronic heart failure: The Task Force for the diagnosis and treatment of acute and chronic heart failure of the European Society of Cardiology (ESC)Developed with the special contribution of the Heart Failure Association (HFA) of the ESC. Eur Heart J. 2016 Jul 14;37(27):2129-2200
23. Piepoli MF, Hoes AW, Agewall S, Albus C, Brotons C, Catapano AL, Cooney MT, Corrà U, Cosyns B, Deaton C, Graham I, Hall MS, Hobbs FDR, Løchen ML, Löllgen H, Marques-Vidal P, Perk J, Prescott E, Redon J, Richter DJ, Sattar N, Smulders Y, Tiberi M, van der Worp HB, van Dis I, Verschuren WMM, Binno S; ESC Scientific Document Group. 2016 European Guidelines on cardiovascular disease prevention in clinical practice: The Sixth Joint Task Force of the European Society of Cardiology and Other Societies on Cardiovascular Disease Prevention in Clinical Practice (constituted by representatives of 10 societies and by invited experts)Developed with the special contribution of the European Association for Cardiovascular Prevention & Rehabilitation (EACPR). Eur Heart J. 2016 Aug 1;37(29):2315-2381.
24. Yancy CW, Jessup M, Bozkurt B, Butler J, Casey DE Jr, Colvin MM, Drazner MH, Filippatos G, Fonarow GC, Givertz MM, Hollenberg SM, Lindenfeld J, Masoudi FA, McBride PE, Peterson PN, Stevenson LW, Westlake C. 2016 ACC/AHA/HFSA Focused Update on New Pharmacological Therapy for Heart Failure: An Update of the 2013 ACCF/AHA Guideline for the Management of Heart Failure: A Report of the American College of Cardiology/American Heart Association Task Force on Clinical Practice Guidelines and the Heart Failure Society of America. J Am Coll Cardiol. 2016 Sep 27;68(13):1476-1488. doi: 10.1016/j.jacc.2016.05.011. Epub 2016 May 20. Erratum in: J Am Coll Cardiol. 2016 Sep 27;68(13):1495.
25. Nishimura RA, Otto CM, Bonow RO, Carabello BA, Erwin JP 3rd, Fleisher LA, Jneid H, Mack MJ, McLeod CJ, O'Gara PT, Rigolin VH, Sundt TM 3rd, Thompson A. 2017 AHA/ACC Focused Update of the 2014 AHA/ACC Guideline for the Management of Patients With Valvular Heart Disease: A Report of the American College of Cardiology/American Heart Association Task Force on Clinical Practice Guidelines. Circulation. 2017 Jun 20;135(25):e1159-e1195.
26. Shen WK, Sheldon RS, Benditt DG, Cohen MI, Forman DE, Goldberger ZD, Grubb BP, Hamdan MH, Krahn AD, Link MS, Olshansky B, Raj SR, Sandhu RK, Sorajja D, Sun BC, Yancy CW. 2017 ACC/AHA/HRS Guideline for the Evaluation and Management of Patients With Syncope: A Report of the American College of Cardiology/American Heart Association Task Force on Clinical Practice Guidelines and the Heart Rhythm Society. Circulation. 2017 Aug 1;136(5):e60-e122.
27. Al-Khatib SM, Stevenson WG, Ackerman MJ, Bryant WJ, Callans DJ, Curtis AB, Deal BJ, Dickfeld T, Field ME, Fonarow GC, Gillis AM, Granger CB, Hammill SC, Hlatky MA, Joglar JA, Kay GN, Matlock DD, Myerburg RJ, Page RL. 2017 AHA/ACC/HRS Guideline for Management of Patients With Ventricular Arrhythmias and the Prevention of Sudden Cardiac Death: A Report of the American College of Cardiology/American Heart Association Task Force on Clinical Practice Guidelines and the Heart Rhythm Society. J Am Coll Cardiol. 2018 Oct 2;72(14):e91-e220.
28. Whelton PK, Carey RM, Aronow WS, Casey DE Jr, Collins KJ, Dennison Himmelfarb C, DePalma SM, Gidding S, Jamerson KA, Jones DW, MacLaughlin EJ, Muntner P, Ovbiagele B, Smith SC Jr, Spencer CC, Stafford RS, Taler SJ, Thomas RJ, Williams KA Sr, Williamson JD, Wright JT Jr. 2017 ACC/AHA/AAPA/ABC/ACPM/AGS/APhA/ASH/ASPC/NMA/PCNA Guideline for the Prevention, Detection, Evaluation, and Management of High Blood Pressure in Adults: A Report of the American College of Cardiology/American Heart Association Task Force on Clinical Practice Guidelines. Circulation. 2018 Oct 23;138(17):e484-e594
29. Valgimigli M, Bueno H, Byrne RA, Collet JP, Costa F, Jeppsson A, Jüni P, Kastrati A, Kolh P, Mauri L, Montalescot G, Neumann FJ, Petricevic M, Roffi M, Steg PG, Windecker S, Zamorano JL, Levine GN; ESC Scientific Document Group; ESC Committee for Practice Guidelines (CPG); ESC National Cardiac Societies. 2017 ESC focused update on dual antiplatelet therapy in coronary artery disease developed in collaboration with EACTS: The Task Force for dual antiplatelet therapy in coronary artery disease of the European Society of Cardiology (ESC) and of the European Association for Cardio-Thoracic Surgery (EACTS). Eur Heart J. 2018 Jan 14;39(3):213-260.
30. Ibanez B, James S, Agewall S, Antunes MJ, Bucciarelli-Ducci C, Bueno H, Caforio ALP, Crea F, Goudevenos JA, Halvorsen S, Hindricks G, Kastrati A, Lenzen MJ, Prescott E, Roffi M, Valgimigli M, Varenhorst C, Vranckx P, Widimský P; ESC Scientific Document Group. 2017 ESC Guidelines for the management of acute myocardial infarction in patients presenting with ST-segment elevation: The Task Force for the management of acute myocardial infarction in patients presenting with ST-segment elevation of the European Society of Cardiology (ESC). Eur Heart J. 2018 Jan 7;39(2):119-177.
31. Baumgartner H, Falk V, Bax JJ, De Bonis M, Hamm C, Holm PJ, Iung B, Lancellotti P, Lansac E, Rodriguez Muñoz D, Rosenhek R, Sjögren J, Tornos Mas P, Vahanian A, Walther T, Wendler O, Windecker S, Zamorano JL; ESC Scientific Document Group. 2017 ESC/EACTS Guidelines for the management of valvular heart disease. Eur Heart J. 2017 Sep 21;38(36):2739-2791.
32. Aboyans V, Ricco JB, Bartelink MEL, Björck M, Brodmann M, Cohnert T, Collet JP, Czerny M, De Carlo M, Debus S, Espinola-Klein C, Kahan T, Kownator S, Mazzolai L, Naylor AR, Roffi M, Röther J, Sprynger M, Tendera M, Tepe G, Venermo M, Vlachopoulos C, Desormais I; ESC Scientific Document Group. 2017 ESC Guidelines on the Diagnosis and Treatment of Peripheral Arterial Diseases, in collaboration with the European Society for Vascular Surgery (ESVS): Document covering atherosclerotic disease of extracranial carotid and vertebral, mesenteric, renal, upper and lower extremity arteriesEndorsed by: the European Stroke Organization (ESO)The Task Force for the Diagnosis and Treatment of Peripheral Arterial Diseases of the European Society of Cardiology (ESC) and of the European Society for Vascular Surgery (ESVS). Eur Heart J. 2018 Mar 1;39(9):763-816.
33. Kusumoto FM, Schoenfeld MH, Barrett C, Edgerton JR, Ellenbogen KA, Gold MR, Goldschlager NF, Hamilton RM, Joglar JA, Kim RJ, Lee R, Marine JE, McLeod CJ, Oken KR, Patton KK, Pellegrini CN, Selzman KA, Thompson A, Varosy PD. 2018 ACC/AHA/HRS Guideline on the Evaluation and Management of Patients With Bradycardia and Cardiac Conduction Delay: A Report of the American College of Cardiology/American Heart Association Task Force on Clinical Practice Guidelines and the Heart Rhythm Society. Circulation. 2019 Aug 20;140(8):e382-e482.
34. Grundy SM, Stone NJ, Bailey AL, Beam C, Birtcher KK, Blumenthal RS, Braun LT, de Ferranti S, Faiella-Tommasino J, Forman DE, Goldberg R, Heidenreich PA, Hlatky MA, Jones DW, Lloyd-Jones D, Lopez-Pajares N, Ndumele CE, Orringer CE, Peralta CA, Saseen JJ, Smith SC Jr, Sperling L, Virani SS, Yeboah J. 2018 AHA/ACC/AACVPR/AAPA/ABC/ACPM/ADA/AGS/APhA/ASPC/NLA/PCNA Guideline on the Management of Blood Cholesterol: A Report of the American College of Cardiology/American Heart Association Task Force on Clinical Practice Guidelines. J Am Coll Cardiol. 2019 Jun 25;73(24):e285-e350.
35. Stout KK, Daniels CJ, Aboulhosn JA, Bozkurt B, Broberg CS, Colman JM, Crumb SR, Dearani JA, Fuller S, Gurvitz M, Khairy P, Landzberg MJ, Saidi A, Valente AM, Van Hare GF. 2018 AHA/ACC Guideline for the Management of Adults With Congenital Heart Disease: A Report of the American College of Cardiology/American Heart Association Task Force on Clinical Practice Guidelines. Circulation. 2019 Apr 2;139(14):e698-e800.
36. Brignole M, Moya A, de Lange FJ, Deharo JC, Elliott PM, Fanciulli A, Fedorowski A, Furlan R, Kenny RA, Martín A, Probst V, Reed MJ, Rice CP, Sutton R, Ungar A, van Dijk JG; ESC Scientific Document Group. 2018 ESC Guidelines for the diagnosis and management of syncope. Eur Heart J. 2018 Jun 1;39(21):1883-1948.
37. Neumann FJ, Sousa-Uva M, Ahlsson A, Alfonso F, Banning AP, Benedetto U, Byrne RA, Collet JP, Falk V, Head SJ, Jüni P, Kastrati A, Koller A, Kristensen SD, Niebauer J, Richter DJ, Seferovic PM, Sibbing D, Stefanini GG, Windecker S, Yadav R, Zembala MO; ESC Scientific Document Group. 2018 ESC/EACTS Guidelines on myocardial revascularization. Eur Heart J. 2019 Jan 7;40(2):87-165.
38. Regitz-Zagrosek V, Roos-Hesselink JW, Bauersachs J, Blomström-Lundqvist C, Cífková R, De Bonis M, Iung B, Johnson MR, Kintscher U, Kranke P, Lang IM, Morais J, Pieper PG, Presbitero P, Price S, Rosano GMC, Seeland U, Simoncini T, Swan L, Warnes CA; ESC Scientific Document Group. 2018 ESC Guidelines for the management of cardiovascular diseases during pregnancy. Eur Heart J. 2018 Sep 7;39(34):3165-3241.
39. Williams B, Mancia G, Spiering W, Agabiti Rosei E, Azizi M, Burnier M, Clement DL, Coca A, de Simone G, Dominiczak A, Kahan T, Mahfoud F, Redon J, Ruilope L, Zanchetti A, Kerins M, Kjeldsen SE, Kreutz R, Laurent S, Lip GYH, McManus R, Narkiewicz K, Ruschitzka F, Schmieder RE, Shlyakhto E, Tsioufis C, Aboyans V, Desormais I; ESC Scientific Document Group. 2018 ESC/ESH Guidelines for the management of arterial hypertension. Eur Heart J. 2018 Sep 1;39(33):3021-3104.
40. January CT, Wann LS, Calkins H, Chen LY, Cigarroa JE, Cleveland JC Jr, Ellinor PT, Ezekowitz MD, Field ME, Furie KL, Heidenreich PA, Murray KT, Shea JB, Tracy CM, Yancy CW. 2019 AHA/ACC/HRS Focused Update of the 2014 AHA/ACC/HRS Guideline for the Management of Patients With Atrial Fibrillation: A Report of the American College of Cardiology/American Heart Association Task Force on Clinical Practice Guidelines and the Heart Rhythm Society. J Am Coll Cardiol. 2019 Jul 9;74(1):104-132.
41. Arnett DK, Blumenthal RS, Albert MA, Buroker AB, Goldberger ZD, Hahn EJ, Himmelfarb CD, Khera A, Lloyd-Jones D, McEvoy JW, Michos ED, Miedema MD, Muñoz D, Smith SC Jr, Virani SS, Williams KA Sr, Yeboah J, Ziaeian B. 2019 ACC/AHA Guideline on the Primary Prevention of Cardiovascular Disease: A Report of the American College of Cardiology/American Heart Association Task Force on Clinical Practice Guidelines. J Am Coll Cardiol. 2019 Sep 10;74(10):e177-e232.
42. Brugada J, Katritsis DG, Arbelo E, Arribas F, Bax JJ, Blomström-Lundqvist C, Calkins H, Corrado D, Deftereos SG, Diller GP, Gomez-Doblas JJ, Gorenek B, Grace A, Ho SY, Kaski JC, Kuck KH, Lambiase PD, Sacher F, Sarquella-Brugada G, Suwalski P, Zaza A; ESC Scientific Document Group. 2019 ESC Guidelines for the management of patients with supraventricular tachycardiaThe Task Force for the management of patients with supraventricular tachycardia of the European Society of Cardiology (ESC). Eur Heart J. 2020 Feb 1;41(5):655-720.
43. Knuuti J, Wijns W, Saraste A, Capodanno D, Barbato E, Funck-Brentano C, Prescott E, Storey RF, Deaton C, Cuisset T, Agewall S, Dickstein K, Edvardsen T, Escaned J, Gersh BJ, Svitil P, Gilard M, Hasdai D, Hatala R, Mahfoud F, Masip J, Muneretto C, Valgimigli M, Achenbach S, Bax JJ; ESC Scientific Document Group. 2019 ESC Guidelines for the diagnosis and management of chronic coronary syndromes. Eur Heart J. 2020 Jan 14;41(3):407-477.
44. Mach F, Baigent C, Catapano AL, Koskinas KC, Casula M, Badimon L, Chapman MJ, De Backer GG, Delgado V, Ference BA, Graham IM, Halliday A, Landmesser U, Mihaylova B, Pedersen TR, Riccardi G, Richter DJ, Sabatine MS, Taskinen MR, Tokgozoglu L, Wiklund O; ESC Scientific Document Group. 2019 ESC/EAS Guidelines for the management of dyslipidaemias: lipid modification to reduce cardiovascular risk. Eur Heart J. 2020 Jan 1;41(1):111-188.
45. Cosentino F, Grant PJ, Aboyans V, Bailey CJ, Ceriello A, Delgado V, Federici M, Filippatos G, Grobbee DE, Hansen TB, Huikuri HV, Johansson I, Jüni P, Lettino M, Marx N, Mellbin LG, Östgren CJ, Rocca B, Roffi M, Sattar N, Seferović PM, Sousa-Uva M, Valensi P, Wheeler DC; ESC Scientific Document Group. 2019 ESC Guidelines on diabetes, pre-diabetes, and cardiovascular diseases developed in collaboration with the EASD. Eur Heart J. 2020 Jan 7;41(2):255-323.
46. Konstantinides SV, Meyer G, Becattini C, Bueno H, Geersing GJ, Harjola VP, Huisman MV, Humbert M, Jennings CS, Jiménez D, Kucher N, Lang IM, Lankeit M, Lorusso R, Mazzolai L, Meneveau N, Ní Áinle F, Prandoni P, Pruszczyk P, Righini M, Torbicki A, Van Belle E, Zamorano JL; ESC Scientific Document Group. 2019 ESC Guidelines for the diagnosis and management of acute pulmonary embolism developed in collaboration with the European Respiratory Society (ERS). Eur Heart J. 2020 Jan 21;41(4):543-603.
47. Ommen SR, Mital S, Burke MA, Day SM, Deswal A, Elliott P, Evanovich LL, Hung J, Joglar JA, Kantor P, Kimmelstiel C, Kittleson M, Link MS, Maron MS, Martinez MW, Miyake CY, Schaff HV, Semsarian C, Sorajja P. 2020 AHA/ACC Guideline for the Diagnosis and Treatment of Patients With Hypertrophic Cardiomyopathy: A Report of the American College of Cardiology/American Heart Association Joint Committee on Clinical Practice Guidelines. J Am Coll Cardiol. 2020 Dec 22;76(25):e159-e240.
48. Otto CM, Nishimura RA, Bonow RO, Carabello BA, Erwin JP 3rd, Gentile F, Jneid H, Krieger EV, Mack M, McLeod C, O'Gara PT, Rigolin VH, Sundt TM 3rd, Thompson A, Toly C. 2020 ACC/AHA Guideline for the Management of Patients With Valvular Heart Disease: A Report of the American College of Cardiology/American Heart Association Joint Committee on Clinical Practice Guidelines. J Am Coll Cardiol. 2021 Feb 2;77(4):e25-e197.
49. Hindricks G, Potpara T, Dagres N, Arbelo E, Bax JJ, Blomström-Lundqvist C, Boriani G, Castella M, Dan GA, Dilaveris PE, Fauchier L, Filippatos G, Kalman JM, La Meir M, Lane DA, Lebeau JP, Lettino M, Lip GYH, Pinto FJ, Thomas GN, Valgimigli M, Van Gelder IC, Van Putte BP, Watkins CL; ESC Scientific Document Group. 2020 ESC Guidelines for the diagnosis and management of atrial fibrillation developed in collaboration with the European Association for Cardio-Thoracic Surgery (EACTS): The Task Force for the diagnosis and management of atrial fibrillation of the European Society of Cardiology (ESC) Developed with the special contribution of the European Heart Rhythm Association (EHRA) of the ESC. Eur Heart J. 2021 Feb 1;42(5):373-498.
50. Collet JP, Thiele H, Barbato E, Barthélémy O, Bauersachs J, Bhatt DL, Dendale P, Dorobantu M, Edvardsen T, Folliguet T, Gale CP, Gilard M, Jobs A, Jüni P, Lambrinou E, Lewis BS, Mehilli J, Meliga E, Merkely B, Mueller C, Roffi M, Rutten FH, Sibbing D, Siontis GCM; ESC Scientific Document Group. 2020 ESC Guidelines for the management of acute coronary syndromes in patients presenting without persistent ST-segment elevation. Eur Heart J. 2021 Apr 7;42(14):1289-1367.
51. Pelliccia A, Sharma S, Gati S, Bäck M, Börjesson M, Caselli S, Collet JP, Corrado D, Drezner JA, Halle M, Hansen D, Heidbuchel H, Myers J, Niebauer J, Papadakis M, Piepoli MF, Prescott E, Roos-Hesselink JW, Graham Stuart A, Taylor RS, Thompson PD, Tiberi M, Vanhees L, Wilhelm M; ESC Scientific Document Group. 2020 ESC Guidelines on sports cardiology and exercise in patients with cardiovascular disease. Eur Heart J. 2021 Jan 1;42(1):17-96.
52. Baumgartner H, De Backer J, Babu-Narayan SV, Budts W, Chessa M, Diller GP, Lung B, Kluin J, Lang IM, Meijboom F, Moons P, Mulder BJM, Oechslin E, Roos-Hesselink JW, Schwerzmann M, Sondergaard L, Zeppenfeld K; ESC Scientific Document Group. 2020 ESC Guidelines for the management of adult congenital heart disease. Eur Heart J. 2021 Feb 11;42(6):563-645.
53. Lawton JS, Tamis-Holland JE, Bangalore S, Bates ER, Beckie TM, Bischoff JM, Bittl JA, Cohen MG, DiMaio JM, Don CW, Fremes SE, Gaudino MF, Goldberger ZD, Grant MC, Jaswal JB, Kurlansky PA, Mehran R, Metkus TS Jr, Nnacheta LC, Rao SV, Sellke FW, Sharma G, Yong CM, Zwischenberger BA. 2021 ACC/AHA/SCAI Guideline for Coronary Artery Revascularization: A Report of the American College of Cardiology/American Heart Association Joint Committee on Clinical Practice Guidelines. Circulation. 2022 Jan 18;145(3):e18-e114.
54. Gulati M, Levy PD, Mukherjee D, Amsterdam E, Bhatt DL, Birtcher KK, Blankstein R, Boyd J, Bullock-Palmer RP, Conejo T, Diercks DB, Gentile F, Greenwood JP, Hess EP, Hollenberg SM, Jaber WA, Jneid H, Joglar JA, Morrow DA, O'Connor RE, Ross MA, Shaw LJ. 2021 AHA/ACC/ASE/CHEST/SAEM/SCCT/SCMR Guideline for the Evaluation and Diagnosis of Chest Pain: A Report of the American College of Cardiology/American Heart Association Joint Committee on Clinical Practice Guidelines. Circulation. 2021 Nov 30;144(22):e368-e454.
55. Glikson M, Nielsen JC, Kronborg MB, Michowitz Y, Auricchio A, Barbash IM, Barrabés JA, Boriani G, Braunschweig F, Brignole M, Burri H, Coats AJS, Deharo JC, Delgado V, Diller GP, Israel CW, Keren A, Knops RE, Kotecha D, Leclercq C, Merkely B, Starck C, Thylén I, Tolosana JM; ESC Scientific Document Group. 2021 ESC Guidelines on cardiac pacing and cardiac resynchronization therapy. Eur Heart J. 2021 Sep 14;42(35):3427-3520.
56. McDonagh TA, Metra M, Adamo M, Gardner RS, Baumbach A, Böhm M, Burri H, Butler J, Čelutkienė J, Chioncel O, Cleland JGF, Coats AJS, Crespo-Leiro MG, Farmakis D, Gilard M, Heymans S, Hoes AW, Jaarsma T, Jankowska EA, Lainscak M, Lam CSP, Lyon AR, McMurray JJV, Mebazaa A, Mindham R, Muneretto C, Francesco Piepoli M, Price S, Rosano GMC, Ruschitzka F, Kathrine Skibelund A; ESC Scientific Document Group. 2021 ESC Guidelines for the diagnosis and treatment of acute and chronic heart failure. Eur Heart J. 2021 Sep 21;42(36):3599-3726.
57. Visseren FLJ, Mach F, Smulders YM, Carballo D, Koskinas KC, Bäck M, Benetos A, Biffi A, Boavida JM, Capodanno D, Cosyns B, Crawford C, Davos CH, Desormais I, Di Angelantonio E, Franco OH, Halvorsen S, Hobbs FDR, Hollander M, Jankowska EA, Michal M, Sacco S, Sattar N, Tokgozoglu L, Tonstad S, Tsioufis KP, van Dis I, van Gelder IC, Wanner C, Williams B; ESC National Cardiac Societies; ESC Scientific Document Group. 2021 ESC Guidelines on cardiovascular disease prevention in clinical practice. Eur Heart J. 2021 Sep 7;42(34):3227-3337.
58. Vahanian A, Beyersdorf F, Praz F, Milojevic M, Baldus S, Bauersachs J, Capodanno D, Conradi L, De Bonis M, De Paulis R, Delgado V, Freemantle N, Gilard M, Haugaa KH, Jeppsson A, Jüni P, Pierard L, Prendergast BD, Sádaba JR, Tribouilloy C, Wojakowski W; ESC/EACTS Scientific Document Group. 2021 ESC/EACTS Guidelines for the management of valvular heart disease. Eur Heart J. 2022 Feb 12;43(7):561-632.
59. Heidenreich PA, Bozkurt B, Aguilar D, Allen LA, Byun JJ, Colvin MM, Deswal A, Drazner MH, Dunlay SM, Evers LR, Fang JC, Fedson SE, Fonarow GC, Hayek SS, Hernandez AF, Khazanie P, Kittleson MM, Lee CS, Link MS, Milano CA, Nnacheta LC, Sandhu AT, Stevenson LW, Vardeny O, Vest AR, Yancy CW. 2022 AHA/ACC/HFSA Guideline for the Management of Heart Failure: A Report of the American College of Cardiology/American Heart Association Joint Committee on Clinical Practice Guidelines. J Am Coll Cardiol. 2022 May 3;79(17):e263-e421.
60. Isselbacher EM, Preventza O, Hamilton Black Iii J, Augoustides JG, Beck AW, Bolen MA, Braverman AC, Bray BE, Brown-Zimmerman MM, Chen EP, Collins TJ, DeAnda A Jr, Fanola CL, Girardi LN, Hicks CW, Hui DS, Jones WS, Kalahasti V, Kim KM, Milewicz DM, Oderich GS, Ogbechie L, Promes SB, Ross EG, Schermerhorn ML, Times SS, Tseng EE, Wang GJ, Woo YJ. 2022 ACC/AHA Guideline for the Diagnosis and Management of Aortic Disease: A Report of the American Heart Association/American College of Cardiology Joint Committee on Clinical Practice Guidelines. J Am Coll Cardiol. 2022 Dec 13;80(24):e223-e393.
61. Zeppenfeld K, Tfelt-Hansen J, de Riva M, Winkel BG, Behr ER, Blom NA, Charron P, Corrado D, Dagres N, de Chillou C, Eckardt L, Friede T, Haugaa KH, Hocini M, Lambiase PD, Marijon E, Merino JL, Peichl P, Priori SG, Reichlin T, Schulz-Menger J, Sticherling C, Tzeis S, Verstrael A, Volterrani M; ESC Scientific Document Group. 2022 ESC Guidelines for the management of patients with ventricular arrhythmias and the prevention of sudden cardiac death. Eur Heart J. 2022 Oct 21;43(40):3997-4126.
62. Halvorsen S, Mehilli J, Cassese S, Hall TS, Abdelhamid M, Barbato E, De Hert S, de Laval I, Geisler T, Hinterbuchner L, Ibanez B, Lenarczyk R, Mansmann UR, McGreavy P, Mueller C, Muneretto C, Niessner A, Potpara TS, Ristić A, Sade LE, Schirmer H, Schüpke S, Sillesen H, Skulstad H, Torracca L, Tutarel O, Van Der Meer P, Wojakowski W, Zacharowski K; ESC Scientific Document Group. 2022 ESC Guidelines on cardiovascular assessment and management of patients undergoing non-cardiac surgery. Eur Heart J. 2022 Oct 14;43(39):3826-3924.
63. Lyon AR, López-Fernández T, Couch LS, Asteggiano R, Aznar MC, Bergler-Klein J, Boriani G, Cardinale D, Cordoba R, Cosyns B, Cutter DJ, de Azambuja E, de Boer RA, Dent SF, Farmakis D, Gevaert SA, Gorog DA, Herrmann J, Lenihan D, Moslehi J, Moura B, Salinger SS, Stephens R, Suter TM, Szmit S, Tamargo J, Thavendiranathan P, Tocchetti CG, van der Meer P, van der Pal HJH; ESC Scientific Document Group. 2022 ESC Guidelines on cardio-oncology developed in collaboration with the European Hematology Association (EHA), the European Society for Therapeutic Radiology and Oncology (ESTRO) and the International Cardio-Oncology Society (IC-OS). Eur Heart J. 2022 Nov 1;43(41):4229-4361.
64. Humbert M, Kovacs G, Hoeper MM, Badagliacca R, Berger RMF, Brida M, Carlsen J, Coats AJS, Escribano-Subias P, Ferrari P, Ferreira DS, Ghofrani HA, Giannakoulas G, Kiely DG, Mayer E, Meszaros G, Nagavci B, Olsson KM, Pepke-Zaba J, Quint JK, Rådegran G, Simonneau G, Sitbon O, Tonia T, Toshner M, Vachiery JL, Vonk Noordegraaf A, Delcroix M, Rosenkranz S; ESC/ERS Scientific Document Group. 2022 ESC/ERS Guidelines for the diagnosis and treatment of pulmonary hypertension. Eur Heart J. 2022 Oct 11;43(38):3618-3731.
65. Joglar JA, Chung MK, Armbruster AL, Benjamin EJ, Chyou JY, Cronin EM, Deswal A, Eckhardt LL, Goldberger ZD, Gopinathannair R, Gorenek B, Hess PL, Hlatky M, Hogan G, Ibeh C, Indik JH, Kido K, Kusumoto F, Link MS, Linta KT, Marcus GM, McCarthy PM, Patel N, Patton KK, Perez MV, Piccini JP, Russo AM, Sanders P, Streur MM, Thomas KL, Times S, Tisdale JE, Valente AM, Van Wagoner DR; Peer Review Committee Members. 2023 ACC/AHA/ACCP/HRS Guideline for the Diagnosis and Management of Atrial Fibrillation: A Report of the American College of Cardiology/American Heart Association Joint Committee on Clinical Practice Guidelines. Circulation. 2024 Jan 2;149(1):e1-e156.
66. Virani SS, Newby LK, Arnold SV, Bittner V, Brewer LC, Demeter SH, Dixon DL, Fearon WF, Hess B, Johnson HM, Kazi DS, Kolte D, Kumbhani DJ, LoFaso J, Mahtta D, Mark DB, Minissian M, Navar AM, Patel AR, Piano MR, Rodriguez F, Talbot AW, Taqueti VR, Thomas RJ, van Diepen S, Wiggins B, Williams MS; Peer Review Committee Members. 2023 AHA/ACC/ACCP/ASPC/NLA/PCNA Guideline for the Management of Patients With Chronic Coronary Disease: A Report of the American Heart Association/American College of Cardiology Joint Committee on Clinical Practice Guidelines. Circulation. 2023 Aug 29;148(9):e9-e119.
67. Byrne RA, Rossello X, Coughlan JJ, Barbato E, Berry C, Chieffo A, Claeys MJ, Dan GA, Dweck MR, Galbraith M, Gilard M, Hinterbuchner L, Jankowska EA, Jüni P, Kimura T, Kunadian V, Leosdottir M, Lorusso R, Pedretti RFE, Rigopoulos AG, Rubini Gimenez M, Thiele H, Vranckx P, Wassmann S, Wenger NK, Ibanez B; ESC Scientific Document Group. 2023 ESC Guidelines for the management of acute coronary syndromes. Eur Heart J. 2023 Oct 12;44(38):3720-3826.
68. Delgado V, Ajmone Marsan N, de Waha S, Bonaros N, Brida M, Burri H, Caselli S, Doenst T, Ederhy S, Erba PA, Foldager D, Fosbøl EL, Kovac J, Mestres CA, Miller OI, Miro JM, Pazdernik M, Pizzi MN, Quintana E, Rasmussen TB, Ristić AD, Rodés-Cabau J, Sionis A, Zühlke LJ, Borger MA; ESC Scientific Document Group. 2023 ESC Guidelines for the management of endocarditis. Eur Heart J. 2023 Oct 14;44(39):3948-4042.
69. Arbelo E, Protonotarios A, Gimeno JR, Arbustini E, Barriales-Villa R, Basso C, Bezzina CR, Biagini E, Blom NA, de Boer RA, De Winter T, Elliott PM, Flather M, Garcia-Pavia P, Haugaa KH, Ingles J, Jurcut RO, Klaassen S, Limongelli G, Loeys B, Mogensen J, Olivotto I, Pantazis A, Sharma S, Van Tintelen JP, Ware JS, Kaski JP; ESC Scientific Document Group. 2023 ESC Guidelines for the management of cardiomyopathies. Eur Heart J. 2023 Oct 1;44(37):3503-3626.
70. Marx N, Federici M, Schütt K, Müller-Wieland D, Ajjan RA, Antunes MJ, Christodorescu RM, Crawford C, Di Angelantonio E, Eliasson B, Espinola-Klein C, Fauchier L, Halle M, Herrington WG, Kautzky-Willer A, Lambrinou E, Lesiak M, Lettino M, McGuire DK, Mullens W, Rocca B, Sattar N; ESC Scientific Document Group. 2023 ESC Guidelines for the management of cardiovascular disease in patients with diabetes. Eur Heart J. 2023 Oct 14;44(39):4043-4140.
71. Ommen SR, Ho CY, Asif IM, Balaji S, Burke MA, Day SM, Dearani JA, Epps KC, Evanovich L, Ferrari VA, Joglar JA, Khan SS, Kim JJ, Kittleson MM, Krittanawong C, Martinez MW, Mital S, Naidu SS, Saberi S, Semsarian C, Times S, Waldman CB. 2024 AHA/ACC/AMSSM/HRS/PACES/SCMR Guideline for the Management of Hypertrophic Cardiomyopathy: A Report of the American Heart Association/American College of Cardiology Joint Committee on Clinical Practice Guidelines. Circulation. 2024 Jun 4;149(23):e1239-e1311.
72. Gornik HL, Aronow HD, Goodney PP, Arya S, Brewster LP, Byrd L, Chandra V, Drachman DE, Eaves JM, Ehrman JK, Evans JN, Getchius TSD, Gutiérrez JA, Hawkins BM, Hess CN, Ho KJ, Jones WS, Kim ESH, Kinlay S, Kirksey L, Kohlman-Trigoboff D, Long CA, Pollak AW, Sabri SS, Sadwin LB, Secemsky EA, Serhal M, Shishehbor MH, Treat-Jacobson D, Wilkins LR. 2024 ACC/AHA/AACVPR/APMA/ABC/SCAI/SVM/SVN/SVS/SIR/VESS Guideline for the Management of Lower Extremity Peripheral Artery Disease: A Report of the American College of Cardiology/American Heart Association Joint Committee on Clinical Practice Guidelines. Circulation. 2024 Jun 11;149(24):e1313-e1410.
73. Van Gelder IC, Rienstra M, Bunting KV, Casado-Arroyo R, Caso V, Crijns HJGM, et al; ESC Scientific Document Group. 2024 ESC Guidelines for the management of atrial fibrillation developed in collaboration with the European Association for Cardio-Thoracic Surgery (EACTS). Eur Heart J. 2024 Aug 30:ehae176
74. Vrints C, Andreotti F, Koskinas KC, Rossello X, Adamo M, Ainslie J, et al; ESC Scientific Document Group. 2024 ESC Guidelines for the management of chronic coronary syndromes. Eur Heart J. 2024 Aug 30:ehae177.
75. McEvoy JW, McCarthy CP, Bruno RM, Brouwers S, Canavan MD, Ceconi C, et al; ESC Scientific Document Group. 2024 ESC Guidelines for the management of elevated blood pressure and hypertension. Eur Heart J. 2024 Aug 30:ehae178.
76. Mazzolai L, Teixido-Tura G, Lanzi S, Boc V, Bossone E, Brodmann M, et al; ESC Scientific Document Group. 2024 ESC Guidelines for the management of peripheral arterial and aortic diseases. Eur Heart J. 2024 Aug 30:ehae179.

**Supplementary Table 5** – Guideline’s methods classification according to Trevisiol et al. 2019.

| **Guideline’s classification (according to Trevisiol et al. 2019)** | | |
| --- | --- | --- |
| **noLA** | Literature Analysis not declared | 0 (0 ESC, 0 ACC/AHA) |
| **noSR** | No declaration of Literature Analysis by Systematic Search | 45 (45 ESC, 0 ACC/AHA) |
| **SRnoDT** | Details of Systematic Search Methods not reported | 3 (2 ESC, 2 ACC/AHA) |
| **SRinsDT** | Methods do not meet predefined quality criteria (*vide* Supplementary Table 2) | 22 (0 ESC, 22 ACC/AHA) |
| **SR** | Methods meet predefined quality criteria (*vide* Supplementary Table 2) | 6 (1 ESC, 5 ACC/AHA) |

**Supplementary Table 6** – AGREE II tool 3^rd^ domain individual categories scoring according to cardiovascular topic.

|  | **ESC** | | | | | | | | | **ACC/AHA** | | | | | | | | |
| --- | --- | --- | --- | --- | --- | --- | --- | --- | --- | --- | --- | --- | --- | --- | --- | --- | --- | --- |
|  | **AGREE II domain 3 (mean±SD)** | | | | | | | | | **AGREE II domain 3 (mean±SD)** | | | | | | | | |
|  | **point 7** | **point 8** | **point 9** | **point 10** | **point 11** | **point 12** | **point 13** | **point 14** | **Over-all** | **point 7** | **point 8** | **point 9** | **point 10** | **point 11** | **point 12** | **point 13** | **point 14** | **Over-all** |
| **Arrhythmias and EP** | 1.3±0.7 | 1.0±0.0 | 2.4±1.0 | 4.0±0.0 | 6.6±0.5 | 6.0±0.0 | 2.0±0.0 | 1.0±0.0 | 24.3±1.5 | 6.3±0.5 | 3.8±2.0 | 4.8±1.3 | 4.2±0.4 | 6.3±0.5 | 6.8±0.4 | 2.2±0.4 | 3.2±1.2 | 36.2±2.1 |
| **CV prevention** | 1.0±0.0 | 1.0±0.0 | 2.3±0.9 | 4.0±0.0 | 6.2±0.4 | 6.0±0.0 | 2.0±0.0 | 1.0±0.0 | 23.6±1.3 | 6.5±0.5 | 5.8±2.2 | 6.0±0.7 | 4.0±0.0 | 6.5±0.5 | 7.0±0.0 | 2.0±0.0 | 1.8±1.3 | 39.5±3.8 |
| **Coronary artery disease** | 1.0±0.0 | 1.0±0.0 | 2.3±0.9 | 4.0±0.0 | 6.4±0.5 | 6.0±0.0 | 2.0±0.0 | 1.0±0.0 | 23.7±1.2 | 5.2±1.2 | 2.4±0.5 | 4.8±1.6 | 4.0±0.0 | 6.4±0.5 | 6.4±0.5 | 2.0±0.0 | 2.4±0.8 | 33.6±1.9 |
| **Heart failure and myocardial disease** | 1.0±0.0 | 1.0±0.0 | 2.0±0.0 | 4.0±0.0 | 6.5±0.5 | 6.0±0.0 | 2.3±0.4 | 1.0±0.0 | 23.8±0.4 | 5.8±2.4 | 2.8±0.5 | 4.4±1.2 | 4.0±0.0 | 6.4±0.5 | 6.8±0.4 | 2.0±0.0 | 2.4±0.8 | 34.2±1.6 |
| **Congenital and valvular heart disease** | 1.9±2.1 | 1.9±2.1 | 2.7+1.7 | 4.0±0.0 | 6.1±0.3 | 6.0±0.0 | 2.0±0.0 | 1.0±0.0 | 25.6±5.9 | 5.3±1.7 | 4.0±2.2 | 5.3±0.5 | 4.0±0.0 | 6.3±0.5 | 7.0±0.0 | 2.0±0.0 | 3.3±0.9 | 37.3±4.0 |
| **General cardiology** | 1.2±0.4 | 1.0±0.0 | 2.0±0.0 | 4.0±0.0 | 6.2±0.4 | 6.0±0.0 | 2.0±0.0 | 1.0±0.0 | 23.3±0.5 | 6.0±0.0 | 3.3±1.2 | 5.3±0.5 | 4.0±0.0 | 6.0±0.0 | 6.7±0.5 | 2.0±0.0 | 3.3±0.9 | 36.7±2.5 |
| **Vascular medicine** | 1.0±0.0 | 1.0±0.0 | 2.8±1.3 | 4.0±0.0 | 6.5±0.5 | 6.0±0.0 | 2.0±0.0 | 1.0±0.0 | 24.3±1.6 | 6.0±0.0 | 2.3±0.5 | 5.0±0.0 | 4.0±0.0 | 6.3±0.5 | 7.0±0.0 | 2.0±0.0 | 2.7±0.9 | 35.3±1.2 |

**Supplementary Table 7** – Characteristics of guidelines excluded due to lack of representativeness (defined as ≤ 20 recommendations).

| **Guideline** | **Soc.** | **Recommendations** | | | | | | | **AGREE II domain 3** | | | | | | | |
| --- | --- | --- | --- | --- | --- | --- | --- | --- | --- | --- | --- | --- | --- | --- | --- | --- |
|  |  | **I** | **IIa** | **IIb** | **III** | **A** | **B** | **C** | **7** | **8** | **9** | **10** | **11** | **12** | **13** | **14** |
| **2014 Focused Update of the Guideline on Stable Ischemic Heart Disease (1)** | ACC/  AHA | 3 | 2 | 3 | 0 | 0 | 3 | 5 | 5 | 3 | 2 | 5 | 6 | 6 | 3 | 2 |
| **2023 Focused Update on the diagnosis and treatment of acute and chronic heart failure (2)** | ESC | 6 | 1 | 0 | 0 | 6 | 1 | 0 | 1 | 1 | 2 | 4 | 7 | 6 | 2 | 1 |
| **2013 Guideline on the Assessment of Cardiovascular Risk (3)** | ACC/  AHA | 1 | 1 | 3 | 1 | 0 | 4 | 2 | 7 | 7 | 7 | 4 | 7 | 7 | 3 | 2 |
| **2013 Guideline on Lifestyle Management to Reduce Cardiovascular Risk (4)** | ACC/  AHA | 7 | 3 | 0 | 0 | 9 | 1 | 0 | 7 | 7 | 7 | 4 | 7 | 7 | 3 | 1 |
| **2013 Guideline for the Management of Overweight and Obesity in Adults (5)** | ACC/  AHA | 10 | 5 | 1 | 0 | 11 | 3 | 2 | 6 | 7 | 7 | 4 | 7 | 7 | 3 | 1 |

References for Supplementary Table 7

1. Fihn SD, Blankenship JC, Alexander KP, Bittl JA, Byrne JG, Fletcher BJ, et al. 2014 ACC/AHA/AATS/ PCNA/SCAI/STS focused update of the guideline for the diagnosis and management of patients with stable ischemic heart disease: a report of the American College of Cardiology/American Heart Association Task Force on Practice Guidelines, and the American Association for Thoracic Surgery, Preventive Cardiovascular Nurses Association, Society for Cardiovascular Angiography and Interventions, and Society of Thoracic Surgeons. J Am Coll Cardiol. 2014 Nov 4;64(18):1929-49
2. McDonagh TA, Metra M, Adamo M, Gardner RS, Baumbach A, Böhm M, et al. 2023 Focused Update of the 2021 ESC Guidelines for the diagnosis and treatment of acute and chronic heart failure. Eur Heart J. 2023 Oct 1;44(37):3627-3639.
3. Goff DC Jr, Lloyd-Jones DM, Bennett G, Coady S, D'Agostino RB, Gibbons R, et al. 2013 ACC/AHA guideline on the assessment of cardiovascular risk: a report of the American College of Cardiology/American Heart Association Task Force on Practice Guidelines. Circulation. 2014 Jun 24;129(25 Suppl 2):S49-73.
4. Eckel RH, Jakicic JM, Ard JD, de Jesus JM, Houston Miller N, Hubbard VS, et al. 2013 AHA/ACC guideline on lifestyle management to reduce cardiovascular risk: a report of the American College of Cardiology/American Heart Association Task Force on Practice Guidelines. Circulation. 2014 Jun 24;129(25 Suppl 2):S76-99.
5. Jensen MD, Ryan DH, Apovian CM, Ard JD, Comuzzie AG, Donato KA, et al. 2013 AHA/ACC/TOS guideline for the management of overweight and obesity in adults: a report of the American College of Cardiology/American Heart Association Task Force on Practice Guidelines and The Obesity Society. Circulation. 2014 Jun 24;129(25 Suppl 2):S102-38.

**Supplementary Figure 1** – PRISMA flowchart.

Records identified (n = 251):

ESC library: n = 34

ACC/AHA libraries: n = 30

Additional PubMed searches: n = 187

Duplicate records removed

(n = 131)

Records screened

(n = 120)

Not full guideline version (n = 31)

76 guidelines (29 ACC/AHA and 47 ESC) included

**Identification of studies via databases**

**Identification**

**Screening**

Full-text articles assessed for eligibility (n = 89)

**Eligibility**

Full-text articles excluded (n=13)

- No summarised recommendations (8);
- <10 recommendations (4);
- 10-20 recommendations (1).

**PRISMA Checklist**

| **Section and Topic** | **Item #** | **Checklist item** | **Location where item is reported** |
| --- | --- | --- | --- |
| **TITLE** | | |  |
| Title | 1 | Identify the report as a systematic review. | 1 |
| **ABSTRACT** | | |  |
| Abstract | 2 | See the PRISMA 2020 for Abstracts checklist. | 3 |
| **INTRODUCTION** | | |  |
| Rationale | 3 | Describe the rationale for the review in the context of existing knowledge. | 5 |
| Objectives | 4 | Provide an explicit statement of the objective(s) or question(s) the review addresses. | 5,6 |
| **METHODS** | | |  |
| Eligibility criteria | 5 | Specify the inclusion and exclusion criteria for the review and how studies were grouped for the syntheses. | 6,7 |
| Information sources | 6 | Specify all databases, registers, websites, organisations, reference lists and other sources searched or consulted to identify studies. Specify the date when each source was last searched or consulted. | 6 |
| Search strategy | 7 | Present the full search strategies for all databases, registers and websites, including any filters and limits used. | 6 |
| Selection process | 8 | Specify the methods used to decide whether a study met the inclusion criteria of the review, including how many reviewers screened each record and each report retrieved, whether they worked independently, and if applicable, details of automation tools used in the process. | 6,7 |
| Data collection process | 9 | Specify the methods used to collect data from reports, including how many reviewers collected data from each report, whether they worked independently, any processes for obtaining or confirming data from study investigators, and if applicable, details of automation tools used in the process. | 7,8 |
| Data items | 10a | List and define all outcomes for which data were sought. Specify whether all results that were compatible with each outcome domain in each study were sought (e.g. for all measures, time points, analyses), and if not, the methods used to decide which results to collect. | 7,8 |
|  | 10b | List and define all other variables for which data were sought (e.g. participant and intervention characteristics, funding sources). Describe any assumptions made about any missing or unclear information. | 7,8 |
| Study risk of bias assessment | 11 | Specify the methods used to assess risk of bias in the included studies, including details of the tool(s) used, how many reviewers assessed each study and whether they worked independently, and if applicable, details of automation tools used in the process. | 7,8 |
| Effect measures | 12 | Specify for each outcome the effect measure(s) (e.g. risk ratio, mean difference) used in the synthesis or presentation of results. | N/A |
| Synthesis methods | 13a | Describe the processes used to decide which studies were eligible for each synthesis (e.g. tabulating the study intervention characteristics and comparing against the planned groups for each synthesis (item #5)). | 7 |
|  | 13b | Describe any methods required to prepare the data for presentation or synthesis, such as handling of missing summary statistics, or data conversions. | 7 |
|  | 13c | Describe any methods used to tabulate or visually display results of individual studies and syntheses. | 7 |
|  | 13d | Describe any methods used to synthesize results and provide a rationale for the choice(s). If meta-analysis was performed, describe the model(s), method(s) to identify the presence and extent of statistical heterogeneity, and software package(s) used. | 7,8 |
|  | 13e | Describe any methods used to explore possible causes of heterogeneity among study results (e.g. subgroup analysis, meta-regression). | 7,8 |
|  | 13f | Describe any sensitivity analyses conducted to assess robustness of the synthesized results. | 7,8 |
| Reporting bias assessment | 14 | Describe any methods used to assess risk of bias due to missing results in a synthesis (arising from reporting biases). | N/A |
| Certainty assessment | 15 | Describe any methods used to assess certainty (or confidence) in the body of evidence for an outcome. | 7,8 |
| **RESULTS** | | |  |
| Study selection | 16a | Describe the results of the search and selection process, from the number of records identified in the search to the number of studies included in the review, ideally using a flow diagram. | 9, S1 |
|  | 16b | Cite studies that might appear to meet the inclusion criteria, but which were excluded, and explain why they were excluded. | 9, S1 |
| Study characteristics | 17 | Cite each included study and present its characteristics. | 9 |
| Risk of bias in studies | 18 | Present assessments of risk of bias for each included study. | 9,10 |
| Results of individual studies | 19 | For all outcomes, present, for each study: (a) summary statistics for each group (where appropriate) and (b) an effect estimate and its precision (e.g. confidence/credible interval), ideally using structured tables or plots. | 9,10 |
| Results of syntheses | 20a | For each synthesis, briefly summarise the characteristics and risk of bias among contributing studies. | 9,10 |
|  | 20b | Present results of all statistical syntheses conducted. If meta-analysis was done, present for each the summary estimate and its precision (e.g. confidence/credible interval) and measures of statistical heterogeneity. If comparing groups, describe the direction of the effect. | 9-11 |
|  | 20c | Present results of all investigations of possible causes of heterogeneity among study results. | N/A |
|  | 20d | Present results of all sensitivity analyses conducted to assess the robustness of the synthesized results. | 11 |
| Reporting biases | 21 | Present assessments of risk of bias due to missing results (arising from reporting biases) for each synthesis assessed. | 10,11 |
| Certainty of evidence | 22 | Present assessments of certainty (or confidence) in the body of evidence for each outcome assessed. | N/A |
| **DISCUSSION** | | |  |
| Discussion | 23a | Provide a general interpretation of the results in the context of other evidence. | 12-14 |
|  | 23b | Discuss any limitations of the evidence included in the review. | 14 |
|  | 23c | Discuss any limitations of the review processes used. | 14,15 |
|  | 23d | Discuss implications of the results for practice, policy, and future research. | 15 |
| **OTHER INFORMATION** | | |  |
| Registration and protocol | 24a | Provide registration information for the review, including register name and registration number, or state that the review was not registered. | 2 |
|  | 24b | Indicate where the review protocol can be accessed, or state that a protocol was not prepared. | 2 |
|  | 24c | Describe and explain any amendments to information provided at registration or in the protocol. | 2 |
| Support | 25 | Describe sources of financial or non-financial support for the review, and the role of the funders or sponsors in the review. | 2 |
| Competing interests | 26 | Declare any competing interests of review authors. | 2 |
| Availability of data, code and other materials | 27 | Report which of the following are publicly available and where they can be found: template data collection forms; data extracted from included studies; data used for all analyses; analytic code; any other materials used in the review. | 2 |

*From:*  Page MJ, McKenzie JE, Bossuyt PM, Boutron I, Hoffmann TC, Mulrow CD, et al. The PRISMA 2020 statement: an updated guideline for reporting systematic reviews. BMJ 2021;372:n71
